# Pancreatic CAF-derived Autotaxin (ATX) drives autocrine CTGF expression to modulate pro-tumorigenic signaling

**DOI:** 10.1158/1535-7163.MCT-23-0522

**Published:** 2025-02-04

**Authors:** Fanny Volat, Ragini Medhi, Lauren Z. Maggs, Marcel A. Deken, Alice Price, Lauren Andrews, Jonathan Clark, Diane Taylor, Alan Carruthers, Ewan Taylor-Smith, Natalia Pacheco, Simon A. Rudge, Amy Fraser, Andrea F. Lopez-Clavijo, Bebiana C. Sousa, Zoë Johnson, Giusy Di Conza, Lars van der Veen, Pritom Shah, Hilary Sandig, Hayley J. Sharpe, Stuart Farrow

**Affiliations:** 1 Cancer Research Horizons, Cambridge, UK; 2 University of Cambridge; 3 The Babraham Institute, Cambridge, UK; 4 iOnctura BV, Amsterdam, the Netherlands; 5 iOnctura SA, Geneva, Switzerland

## Abstract

Autotaxin (ATX), encoded by *ENPP2*, is a clinical target in pancreatic ductal adenocarcinoma (PDAC). ATX catalyzes the production of lysophosphatidic acid (LPA), an important regulator within the tumor microenvironment (TME), yet the pro-tumorigenic action of the ATX/LPA axis in PDAC remains unclear. Here, by interrogating patient samples and cell line datasets, we show that the PDAC TME, rather than cancer cells, is responsible for the majority of *ENPP2* expression, and highlight a key role for cancer associated fibroblast (CAF)-derived ATX in autocrine and paracrine pro-tumorigenic signaling. Using the clinical-stage ATX inhibitor, IOA-289, we identified connective tissue growth factor (CTGF), also known as CCN2, as a downstream mediator of ATX signaling in the PDAC CAF-derived cell line, 0082T. Genetic ablation or pharmacological inhibition of ATX in 0082T CAFs reduced CTGF secretion via modulation of LPA/LPA receptor (LPAR) signaling. Despite the loss of ATX function, extracellular levels of LPA were paradoxically increased, indicating a role for ATX beyond its enzymatic activity and suggesting a role for its LPA chaperone function in the LPA/LPAR signaling in CAFs. As CAFs are the main source for CTGF in the PDAC TME, these findings suggest a role for ATX in promoting pro-tumorigenic microenvironment via modulation of CAF secretion, not only via its LPA-producing activity but also via its LPA chaperone function, providing a potential mechanism for the anti-tumor effects of ATX inhibition.

## Introduction

Autotaxin (ATX), also known as ENPP2, is a secreted lysophospholipase D enzyme that converts lysophosphatidylcholine (LPC) to lysophosphatidic acid (LPA) (1). Increased ATX and LPA are found in the serum of cancer patients of diverse indications including pancreatic, liver or breast (2–4). Independent studies have shown that ATX promotes several aspects of tumorigenesis such as proliferation, invasion, and cell migration (5), highlighting ATX as a promising target for cancer therapy. The pro-tumorigenic signaling cascades induced by ATX are triggered by its product LPA upon binding to the six LPA receptors, LPAR1-6 (6,7).

We developed the oral, potent, and selective ATX inhibitor, IOA-289 (formerly CRT0273750)(8), which is safe and well-tolerated, while reducing the circulating levels of LPA in the blood of healthy human volunteers (9). In orthotopic mouse models of pancreatic cancer, a combination treatment of IOA-289 with standard-of-care treatment, gemcitabine, significantly reduces tumor volume and favors overall survival (10). Whilst ATX inhibitors have been developed for organ-specific fibrosis indications such as idiopathic pulmonary fibrosis (IPF), IOA-289 is the first ATX inhibitor to enter clinical trials in oncology. IOA-289 is currently under investigation in a Phase Ib clinical trial for the treatment of patients with metastatic pancreatic cancer (NCT05586516).

Pancreatic ductal adenocarcinoma (PDAC) is an aggressive cancer which accounts for most pancreatic cancer cases and has a poor 5-year survival rate of between 2-9% (11). PDAC is characterized by a highly fibrotic tumor microenvironment (TME), accounting for up to 90% of the tumor volume (12), acting as a barrier to therapy and contributing to the poor survival of PDAC patients (13). The fibrotic nature of the PDAC TME is due to an abundance of pancreatic cancer associated fibroblasts (CAFs) depositing a dense extracellular matrix (ECM) (14). Using *in cellulo* models of human pancreatic CAFs, Auciello *et al*., 2019 showed that CAF secretions (containing LPC, LPA and ATX) promote PDAC growth and migration through LPA-LPAR mediated signaling, which are inhibited upon treatment with the ATX inhibitor, HA130 (15). However, the mechanism by which ATX inhibition influences CAFs and their secretome is still not fully characterized.

ATX catalyzes the hydrolysis of the choline head group of LPC, producing an LPA molecule with the same acyl chain length and saturation as the substrate LPC (1). Whilst ATX does not act on the acyl chains, acyl chain length and saturation (i.e. the species) of the LPC impacts its use as an ATX substrate (16). Additionally, LPA species differentially activate LPA receptors and, therefore, differences in LPA species may impact LPAR signaling in the PDAC TME (17).

In this study, we investigate the cellular source of ATX in PDAC and LPA metabolism by PDAC CAFs and tumor cells in the context of pharmacological inhibition of ATX by IOA-289 and genetic ablation. We characterized the impact of ATX inhibition by IOA-289 on both autocrine and paracrine pro-tumorigenic signaling of 0082T CAF cells.

## Materials and Methods

### Cell line maintenance

### *ENPP2* knockout in 0082T cells using CRISPR-Cas9

To form ribonucleoprotein (RNP) complexes, 104 pmol Cas9 protein (Alt-R S.p. Cas9 Nuclease V3, Integrated DNA Technologies (IDT), #1081059) and 120 pmol sgRNAs in 5 μL/reaction were incubated 20 minutes at room temperature (RT). Two sgRNA guides were used to target *ENPP2* (sgENPP2-1: Hs.Cas9.ENPP2.1.AA, sgENPP2-2: Hs.Cas9.ENPP2.1.AB, IDT) and compared to a non-targeting control (NTC) sgRNA (5’ CGTTAATCGCGTATAATACG 3’, IDT). 2.5x10^5^ 0082T cells per nucleofection reaction were spun down at 90xg for 5 min. Cells were resuspended in 20 μL/reaction of Lonza nucleofection SE solution (Lonza, #V4XC-1032) supplemented with electroporation enhancer at 4 μmol/L (IDT, #1075916). The cell solution was then added to the 5 μL of RNP complex and mixed. Cell/RNP mix was then immediately loaded into the 16-well nucleocuvette and nucleofected using CM-137 pulse of the Lonza 4D Nucleofector (RRID:SCR_023155). After nucleofection cells were transferred to a culture plate in complete media for amplification.

### Cell treatment and conditioned media generation (CM)

For LC-MS/MS analysis, cells were seeded at 2x10^5^ cells/well in 6-well plates. The following day, the wells were washed with serum free (SF) DMEM and treated for 48 hours with 1 ml SF DMEM supplemented with 0.5% fatty acid-free bovine serum albumin (FAF BSA) and either with 0.1% DMSO, 12 μmol/L of IOA-289 (8) or 12 μmol/L PF-8380 (18). IOA-289 and PF-8380 compounds were synthesized in-house as detailed in Supplementary Materials and Methods. Following conditioning, supernatants from replicates were collected, pooled, and centrifuged to remove cell debris. After homogenization, supernatants were equally split, one was placed on dry ice and frozen immediately (no CFI) and the other incubated at 37°C for 24 hours before freezing immediately on dry ice (CFI). In experiments with *ENPP2* KO and NTC 0082T cells, a sample of each CM was collected for ATX and connective tissue growth factor (CTGF/CCN2) detection and cells were lysed for gene expression analysis by RT-qPCR.

For the other analyses, cells were seeded 3.5-4x10^4^ cells/cm^2^ in complete media. The following day, cells were washed with SF DMEM (1mM glucose, 0.5mM glutamine) as previously described (15), and 160-300 μl/cm^2^ of SF DMEM supplemented with 0.5% FAF BSA, and without or with 0.1% DMSO, 12 μmol/L of IOA-289 or 12 μmol/L Ki16425 (Sigma-Aldrich Cat# SML0971), was added for 48 hours. CM were collected for ATX and CTGF detection and cell lysates for gene expression analysis by RT-qPCR. For RNA-seq analysis, cells were treated only for 24 hours.

### Folch-butanol lysolipid extraction and analysis from CM

Our previously published method (19) adapted for use with 1 ml of CM by doubling the volume of solvents was used. In the initial Folch extraction, 1 ml of CM takes the place of 500 μL MilliQ water. Samples were analyzed on a Kinetex EVO C18 column using a Sciex QTRAP mass spectrometer. For more detailed methods see Supplementary Materials and Methods.

### RNA extraction and bulk RNA-seq analysis

RNA was extracted from cell lysates using the RNeasy Plus Mini Kit (Qiagen, Cat#74134) according to manufacturer’s protocol and quantified by Nanodrop ND-1000. The samples were diluted with RNase free water to ˜500 ng/μL. Sequencing library preparation and analysis was performed by Genewiz with their standard RNA-seq analysis pipeline. Libraries were prepared with poly-A selected RNA from total RNA. Illumina NovaSeq with 2X150 bp configuration was used for sequencing. DESeq2 (RRID:SCR_015687) (20) was used to identify differentially expressed genes between conditions and Wald test was used to generate p-values.

### Western blots

Denatured CM (50 μL) or cell lysates (40 μg) were loaded and run on a 3-8% Tris-acetate gel and proteins were transferred on nitrocellulose membranes. Membranes were incubated with anti-ATX (MBL International Cat#D323-3, RRID:AB_2819353) and anti-α-Tubulin (Sigma-Aldrich Cat#T9026, RRID:AB_477593) antibodies, overnight at 4°C. Secondary antibody incubations were performed for 1 hour at RT (Licor Cat#926-32219 and Cat#926-68070). Ponceau S was used for total protein stain. Membranes were scanned with LI-COR Odyssey M.

### RT-qPCR

RNA was isolated as described for RNA-seq. RNA was reverse transcribed using High-Capacity cDNA Reverse Transcription Kit (ThermoFisher Cat#4374967), and qPCR was performed from 1 μL cDNA using TaqMan Fast Advanced Master Mix and Taqman probes listed in Supplementary Table S1. Samples were run and analyzed with the Applied Biosystems ViiA 7 Real-Time PCR System (RRID:SCR_023358). For untreated samples, gene expression was presented as absolute expression relative to housekeeping genes *HPRT1, GAPDH* or *RPL13A* (2^-ΔCT^). For compound treated samples and *ENPP2* KO samples, relative gene expression to DMSO and NTC control (2^-ΔΔCT^) was presented.

### ELISA

CM generated from treated or untreated samples were used for ELISA. Human CTGF/CCN2 DuoSet ELISA (R&D Cat#DY9190-05) and DuoSet ELISA Ancillary Reagent kit 2 (R&D Cat#DY008B) were used according to the manufacturer’s guidelines.

### Data handling – Publicly available data

mRNA expression data for the comparison of *ENPP2* expression in primary tumors and normal tissue in all cancers, and the additional matched normal tissue data, comes from combined cohort of TCGA (The Cancer Genome Atlas), TARGET (Therapeutically Applicable Research to Generate Effective Treatments) and GTEx (The Genotype-Tissue Expression) samples, available through the UCSC (University of California, Santa Cruz) Xena Browser (https://xenabrowser.net/) (21). The gene expression data across cancer cell lines comes from the Cancer Cell Line Encyclopedia (CCLE) from the Broad Institute and Novartis, updated 2019 (22,23) and was accessed through cBioPortal (RRID:SCR_014555) (24,25). The pancreatic scRNA-seq dataset from (26) was obtained from zenodo (https://zenodo.org/record/6024273). Seurat (27)was used to query the scRNA-seq dataset. Ucell (28) was used for scRNA-seq dataset scoring and ESTIMATE gene-set validation. Expressing cells defined as cells with non-zero gene expression value.

### Estimate analysis

ESTIMATE (Estimation of Stromal and Immune cell in Malignant Tumor tissues using Expression data) scores of tumor purity were downloaded from https://bioinformatics.mdanderson.org/estimate/ for every available cancer type for the RNA-Seq-V2 platform. cBioPortal (RRID:SCR_014555) was used for handling and downloading TCGA data (24,25). Of the 184 samples in the Pancreatic Adenocarcinoma (TCGA, PanCancer Atlas) dataset, 31 were annotated as non-PDAC (i.e., Pancreatic Adenocarcinoma, other subtype, or Pancreatic Carcinoma) and were removed from the analysis. Similar measures were not taken for the other cancer types studied. TME rich samples were defined as samples with both highest quartile stromal and highest quartile immune scores, and tumor rich samples were defined as samples with both lowest quartile stromal and lowest quartile immune scores (within each cancer type). These groups were compared using cBioPortal. The RSEM (Batch normalized from Illumina HiSeq_RNASeqV2) (log2) data was downloaded for *ENPP2* and *ACTA2* mRNA expression.

## Results

### ATX is primarily expressed by the TME in PDAC tumors

**Figure 1:**
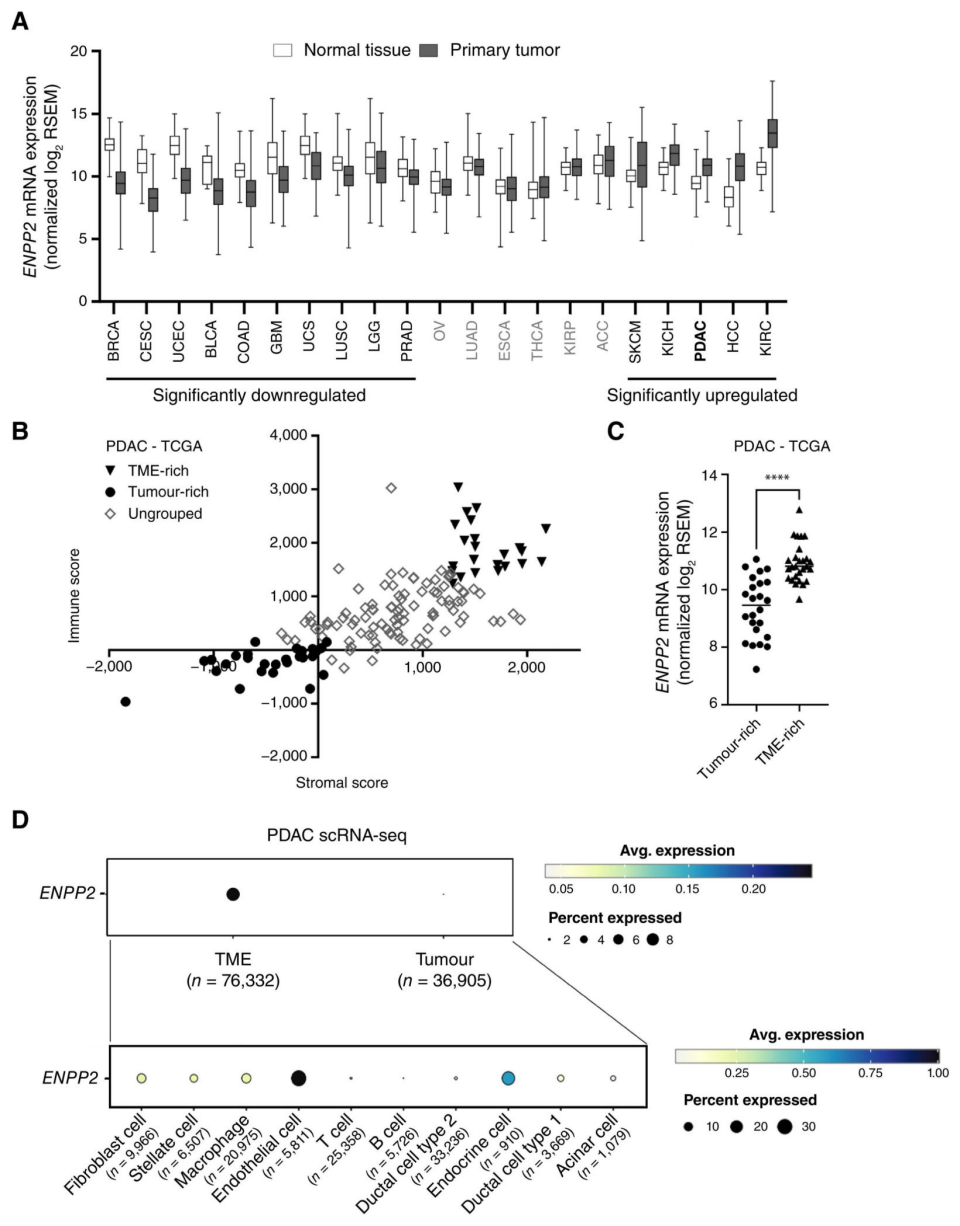
Supporting cells of the PDAC TME, such as CAFs, are the main contributors of ATX. **A**, *ENPP2* mRNA expression (normalised log2 RSEM) in normal tissue compared to primary tumour across several cancer types, displayed in order of difference in *ENPP2* expression. RSEM: RNA-Seq by Expectation-Maximization. Data from UCSC Xena TCGA TARGET GTEx (21). Significance determined by two-way ANOVA and defined as a *p* value <0.05. RSEM: RNA-Seq by Expectation-Maximization. **B**, Grouping of PDAC TCGA samples by ESTIMATE scores (29). Tumour-rich samples (circles) were defined as lowest quartile immune and lowest quartile stromal and TME-rich samples (triangles) were defined as those with highest quartile stromal and highest quartile immune scores. Other samples were labelled as ‘ungrouped’. **C**, *ENPP2* expression in tumour-rich (n=24) and TME-rich (n=27) samples within the TCGA pancreatic cancer data set. Significance determined by t-test, ‘****’ indicates *p* value <0.0001. **D**, *ENPP2* expression in cells of TME and tumour cells in the pancreatic scRNA-seq dataset from (26). *ENPP2-*expressing cells defined as cells with non-zero *ENPP2* expression value. Number of cells in TME or tumour compartment highlighted by ‘n’. Cancer abbreviations as follows: ACC-Adrenocortical Carcinoma; BLCA-Bladder Cancer; BRCA-Breast Invasive carcinoma; CESC-Cervical squamous cell carcinoma and endocervical adenocarcinoma; COAD-Colon adenocarcinoma; ESCA-Esophageal carcinoma; GBM-Glioblastoma multiforme; HCC-Hepatocellular Carcinoma; KICH-Kidney Chromophobe; KIRC-Kidney renal clear cell carcinoma; KIRP-Kidney renal papillary cell carcinoma; LGG- Low grade glioma; LUAD-Lung adenocarcinoma; LUSC-Lung squamous cell carcinoma; OV-Ovarian serous cystadenocarcinoma; PDAC-Pancreatic ductal adenocarcinoma; PRAD-Prostate adenocarcinoma; SKCM-Skin Cutaneous Melanoma; THCA-Thyroid carcinoma, UCS-Uterine Carcinosarcoma; UCEC-Uterine Corpus Endometrial Carcinoma.

ATX, encoded by *ENPP2*, is expressed in multiple tumor types, and PDAC is one of five cancers where *ENPP2* expression is significantly increased in tumor compared to normal tissue (Figure 1A). To explore the source of *ENPP2* expression in PDAC tumors, tumor-rich and TME-rich PDAC human tumor samples were identified in The Cancer Genome Atlas (TCGA) using ‘Estimation of Stromal and Immune cells in Malignant Tumors using Expression data’ (ESTIMATE) scores (29) (Figure 1B). We validated the stromal and immune gene set used in ESTIMATE to assign scores using a single cell RNA sequencing (scRNA-seq) dataset (26) (Figure S1A) and confirmed the increased expression of *ACTA2*, encoding the CAF marker alpha smooth muscle actin (αSMA), in TME-rich samples in all solid tumors studied (Figure S1B). *ENPP2* expression was enriched in TME-rich samples suggesting that the TME, rather than the tumor cells, may be the source of ATX (Figure 1C). Similar enrichment of *ENPP2* expression in the TME was observed in many other cancers, but notably not hepatocellular carcinoma (HCC) and kidney renal clear cell carcinoma (KIRC), which also display *ENPP2* overexpression compared to normal tissue (Figure 1A, S1B-C). The pancreatic scRNA-seq dataset (26) confirmed that *ENPP2* expressing cells were mostly observed in the TME population (including CAF, macrophages, and endothelial cells) rather than the tumor cell population (Figure 1D). Since CAFs are the most abundant cell type in the PDAC TME (11), we focused on their role in ATX signaling.

### 0082T cells display a CAF phenotype and express ATX in the basal state, unlike PDAC cancer cells

**Figure 2:**
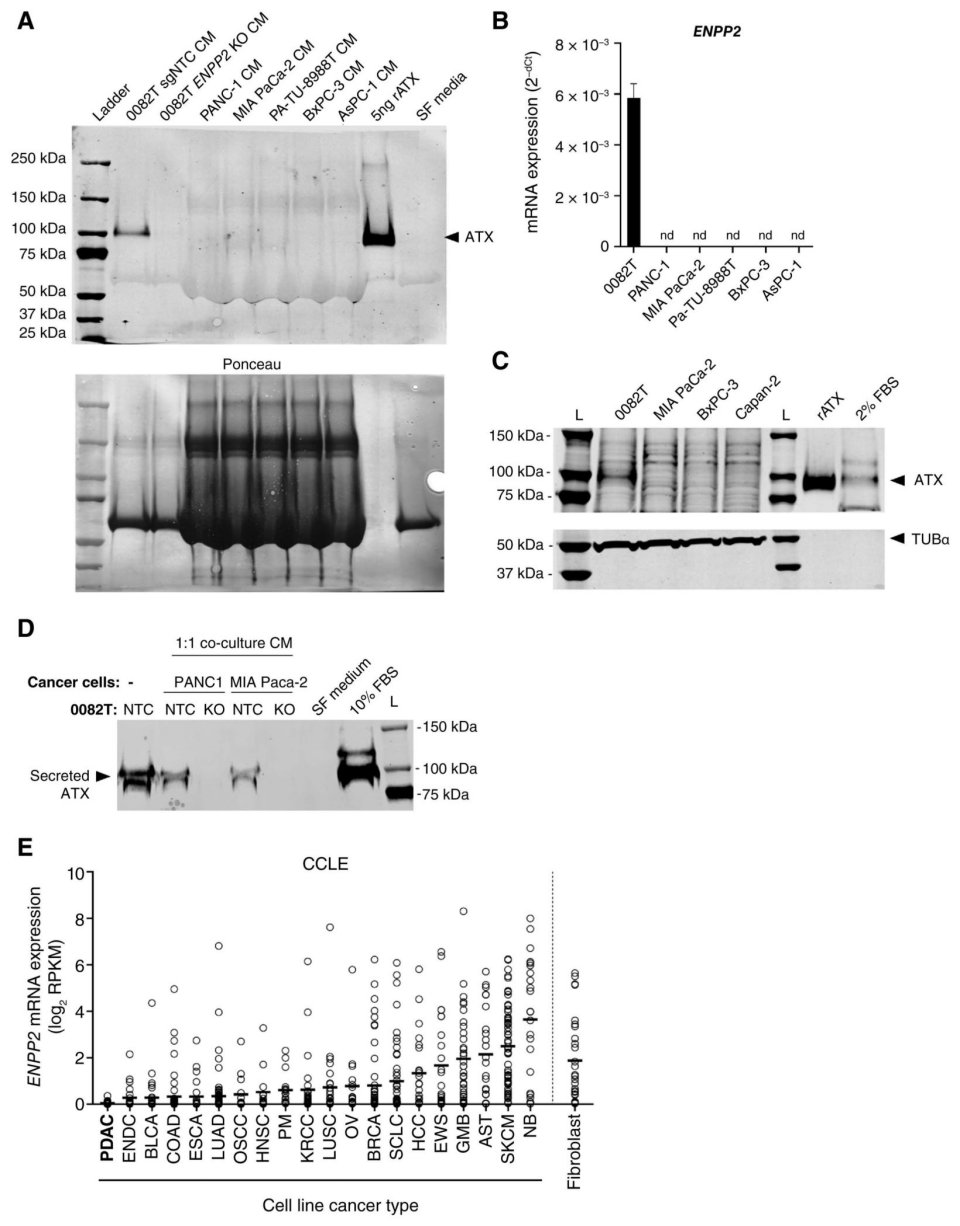
ATX expression in cancer and fibroblasts cell lines. **A**, ATX (˜100 kDa) detection by western blot in the CM generated from the CAF cell line 0082T, NTC (non-targeting control) and *ENPP2* KO, and PDAC cell lines PANC-1, MIA PaCa-2, PA-TU-8988T, BxPC-3 and AsPC-1 cultured in serum free (SF) condition for 48 hours. Recombinant Autotaxin (rATX) and SF DMEM are used as controls. Representative western blot of N=3 independent biological replicates. Ponceau staining is shown as a loading control. **B**, *ENPP2* mRNA expression in 0082T CAFs and PDAC cell lines cultured in SF condition for 48 hours. *RPL13A* was used as housekeeping gene. N=3 biological replicates. **C**, ATX detection by western blot in the cell lysates from the CAF cell line 0082T and PDAC cell lines MIA PaCa-2, BxPC-3 and Capan-2 cultured in their respective complete media for 48 hours. Recombinant Autotaxin (rATX) and 2% FBS DMEM are used as controls. αTubulin is used as a loading control. **D**, ATX detection by western blot in CM generated from co-culture of CAF 0082T (NTC or *ENPP2* KO) with PDAC cell lines (PANC-1 or MIA PaCa-2) at a 1:1 ratio in SF condition for 48 hours. SF medium and 10% FBS DMEM are used as controls. Representative western blot of N=3 independent biological replicates performed with 3 independent 0082T KO pools. **E**, *ENPP2* expression (log2RPKM – RNA-seq) in cancer cell lines derived from the most common cancer types (ranked by mean expression) and in fibroblasts cell lines. Datasets From the cancer cell line encyclopaedia (CCLE). Cancer cell line abbreviations and numbers : AST – Astrocytoma (n=23); BLCA-Bladder Cancer (n=35); BRCA-Breast Invasive carcinoma (n=53); COAD-Colon adenocarcinoma (n=60); ENDC – Endometrial carcinoma (n=24); ESCA-Esophageal carcinoma (n=25); EWS- Ewing Sarcoma (n=25); GBM-Glioblastoma multiforme (n=34); HCC-Hepatocellular Carcinoma (n=23); HNSC - Head and Neck squamous cell carcinoma (n=14); KRCC – Kidney renal cell carcinoma (n=31); LUAD-Lung adenocarcinoma (n=79); LUSC-Lung squamous cell carcinoma (n=27); NB-Neuroblastoma (n=23); OSCC-Oral squamous cell carcinoma (n=15); OV-Ovarian serous cystadenocarcinoma (n=17); PDAC-Pancreatic ductal adenocarcinoma (n=46); PM – Pleural mesothelioma (n=17); SCLC- Small cell lung cancer (n=51); SKCM-Skin Cutaneous Melanoma (n=57). nd, not detected. KO, *ENPP2* knockout using sgRNA-2. NTC, Non-targeting control. L, Ladder. RPKM: Reads Per Kilobase of transcript, per Million mapped reads

To explore the role of ATX in CAFs, we used the previously established human 0082T pancreatic CAF cell line that lacks *KRAS* exon 2 mutations (15). As expected, 0082T cells expressed *ACTA2* and *COL1A1* genes and showed collagen type I deposition in a Scar-in-a-Jar assay compared to PDAC cancer cell lines (Figure S2A-C). We validated the CAF phenotype by confirming that 0082T cells express a high level of αSMA at basal state, in contrast to the requirement for TGF-β1-induced myofibroblast differentiation for normal fibroblasts (Figure S2D,E). To explore ATX secretion by 0082T CAFs, cells were cultured in serum free (SF) medium supplemented with 0.5% FFA BSA as ATX is abundant in FBS (Figure S3A) and conditioned media (CM) were collected. As shown in Figure 2A, ATX was detected in CM from pancreatic CAF 0082T cells but not in CM from the PDAC cell lines studied. The absence of *ENPP2* expression in PDAC cells was confirmed by RT-qPCR (Figure 2B). Since the absence of FBS may influence ATX expression, the absence of ATX was also confirmed in cell lysates from PDAC cancer cells cultured in complete medium (Figure 2C).

The finding that PDAC cell lines do not express ATX is in contrast to published work (15), therefore we used CRISPR KO to confirm the specificity of the antibody we used to detect ATX (Figure 2B and S3A). In addition, we confirmed the absence of ATX in CM when PDAC cells were co-cultured with *ENPP2* KO 0082T at a 1:1 cell ratio (Figure 2D and S3B). In line with these results and our analysis of patient data (Figure 1), PDAC cancer cell lines (n=46) reported in the CCLE database display the lowest *ENPP2* mRNA expression when compared across different cancer and fibroblasts cell lines (Figure 2E). These findings indicate that in *in cellulo* context, PDAC cell lines do not express ATX or express it a very low level (non-detectable with available technologies). However, this does not exclude that within the TME, pancreatic cancer cells might upregulate ATX expression when exposed to stress stimuli (30–33).

### *ATX/LPA signaling regulates* paracrine and autocrine CAF pro-tumorigenic signaling

**Figure 3:**
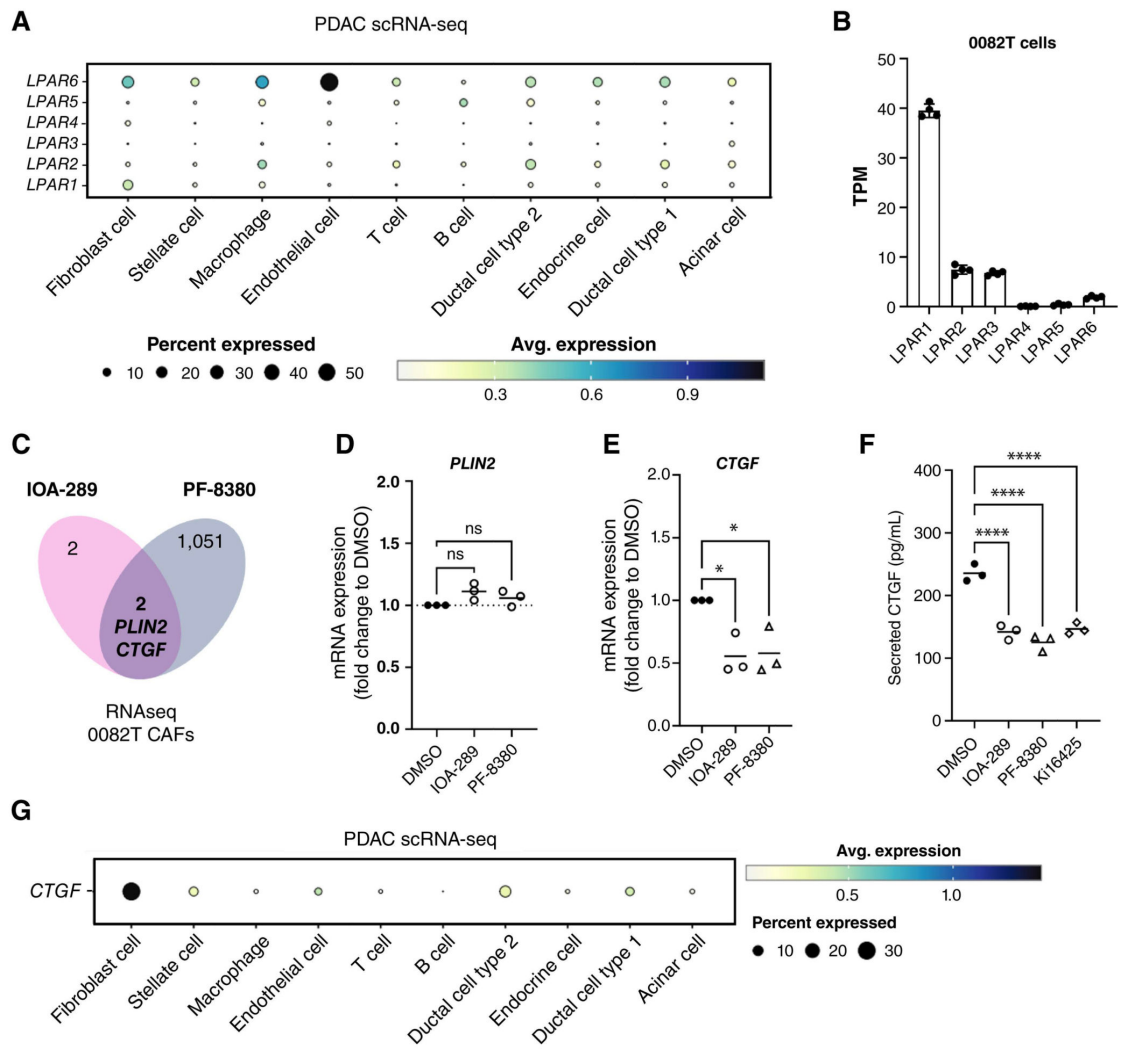
ATX inhibition modulates CTGF expression in PDAC 0082T CAFs via LPAR signaling. **A**, *LPAR1-6* expression in cells of TME and tumour cells in the pancreatic scRNA-seq dataset from (26). **B**, LPA receptors expression in transcript per million (TPM) in 0082T cells treated with 0.1% DMSO for 24 hours (N=4). **C**, Venn diagram from 0082T RNA-seq data showing overlap of significant differentially expressed genes (adjusted p value by Wald test of <0.05) between 0.1% DMSO vs 12 μmol/Lol/L IOA-289 and 0.1% DMSO vs 12 μmol/Lol/L PF-8380 treatments. **D-E**, Relative mRNA expression of *PLIN2* (**D**) and *CTFG* (**E**) in 0082T cells treated with 0.1% DMSO (vehicle control), 12 μmol/L IOA-289 or 12 μmol/L PF-8380 for 48 hours (N=3). *RPL13A* was used as housekeeping gene. Significance determined by one-way ANOVA and post-hoc Dunnett comparisons, “*” indicates *p* value <0.05. **F**, CTGF secretion by 0082T cells treated with 0.1% DMSO, 12 μmol/Lol/L IOA-289, 12 μmol/Lol/L PF-8380, or 12 μmol/Lol/L LPAR1-3 antagonist Ki16425 for 48 hours, measured by ELISA (N=3). Significance determined by one-way ANOVA and post-hoc Dunnett comparisons, “****” indicates *p* value <0.0001. **G**, *CTGF* expression in cells of TME and tumour cells in the pancreatic scRNA-seq dataset from (26).

To investigate the impact of inhibition of the ATX/LPA signaling within the PDAC TME, we first assessed the expression profile of LPARs in the different PDAC cell populations. Exploration of the pancreatic scRNA-seq dataset showed that all cell types in the pancreatic TME express LPARs but with different expression patterns (Figure 3A). In the PDAC TME, CAFs predominantly express *LPAR1* and *LPAR6* (Figure 3A), while *LPAR1* shows the highest levels in 0082T CAF (Figure 3B). In PDAC cell lines and in pancreatic tumor cells *LPAR4* is lowly expressed (Figure 3A and S4A-D) and MIA PaCa-2, PANC-1, PA-TU-8988T, BxPC-3 and AsPC-1 PDAC cells all show distinct LPAR expression profiles (Figure S4B). These findings indicate that CAF-derived ATX/LPA signaling has the potential to act in both an autocrine and paracrine manner in the PDAC TME and to regulate distinct signaling pathways.

Longitudinal PDAC growth studies in presence of CM from 0082T CAF cells were hampered by the need for serum-free conditions to remove exogenous ATX. However, as previously demonstrated (15), short-term treatment of PDAC cells with 0082T concentrated CM consistently increased the confluence of both MIA PaCa-2 and PANC-1 cancer cells (Figure S4E,F). This effect was reduced when CM was generated from 0082T treated with the ATX inhibitor IOA-289 (Figure S4G,H), indicating that ATX regulates 0082T paracrine pro-tumorigenic signaling. To rule out ATX-independent effects of IOA-289, we verified that CM from PANC-1 cells (which do not produce ATX) treated or not with IOA-289 had no impact on MIA PaCa-2 confluency (Figure S4I). Importantly, and unlike the ATX inhibitors PF-8380 and ziritaxestat (GLPG1690) (18,34), IOA-289 did not affect 0082T CAF cell viability (Figure S4J). Interestingly, however, the addition of 18:1 LPA, at a high concentration did not recapitulate the increase of PDAC confluency induced by 0082T CM (Figure S4K). This suggested that different LPA species, a more complex LPA-signaling mechanism and/or another CAF-derived mediator dependent on ATX-LPA signaling was driving the CAF pro-tumorigenic effect.

### ATX inhibition modulates pro-fibrotic CTGF expression and secretion in pancreatic CAF cells

To identify ATX regulated pathways in CAFs other than LPA, we took an unbiased approach and investigated gene expression changes using RNA-seq in 0082T CAF cells treated with or without the ATX inhibitors IOA-289 or PF-8380 for 24 hours. Principal component analysis (PCA) distinguished the differences between PF-8380 treatments and control but not between IOA-289 treatments and control (Figure S5A-B). Indeed, PF-8380 treatment resulted in a higher number of differentially expressed genes compared to IOA-289 treatment at the timepoint tested (Figure 3C) that could be related to the higher levels of cell death observed with the PF-8380 treatment on 0082T CAFs (Figure S4J). IOA-289 treatment resulted in only four significantly differentially expressed genes (*CTGF/CCN2, PLIN2, HHIP*, and *AHNAK)*. However, only *PLIN2*, which was upregulated and *CTGF*, which was downregulated, overlapped between the two ATX inhibitors (Figure 3C and S5C-F). The modulation of *CTGF* expression, but not that of *PLIN2*, upon ATX inhibition was confirmed by qPCR (Figure 3D,E). CTGF, also known as CCN2, is a pro-fibrotic factor that promotes pancreatic tumor growth (35,36). To determine whether the change in gene expression influenced the CAF secretome, CTGF secretion was investigated in 0082T CM after treatment with ATX inhibitors or the LPAR antagonist Ki16425, which inhibits LPAR1 and LPAR3 and, to a lesser extent, LPAR2 (37). All three inhibitors significantly decreased CTGF secretion from 0082T cells (Figure 3F), indicating that ATX regulates CTGF secretion by CAFs via LPA-LPAR signaling. Interestingly, CAFs appear as the key source for CTGF in the pancreatic TME (Figure 3G), therefore we propose that CAF-derived ATX contributes to tumor growth by regulating CTGF secretion in the PDAC TME via autocrine LPA-LPAR signaling.

### Inhibition of CAF-derived ATX increases extracellular levels of LPA

To better understand how ATX regulates CTGF secretion by CAFs, we explored the extracellular lysophospholipids in the CM from 0082T and PANC-1 cells (which do not express ATX) using LC-MS/MS. To capture only the activity of secreted ATX, media containing FBS were replaced with FBS-free media supplemented with 0.5% FAF BSA the day after seeding. To quantify residual lysolipids from the seeding media, a mock condition of an empty well was included (treated in the same way as the wells with cells) and used as control.

**Figure 4:**
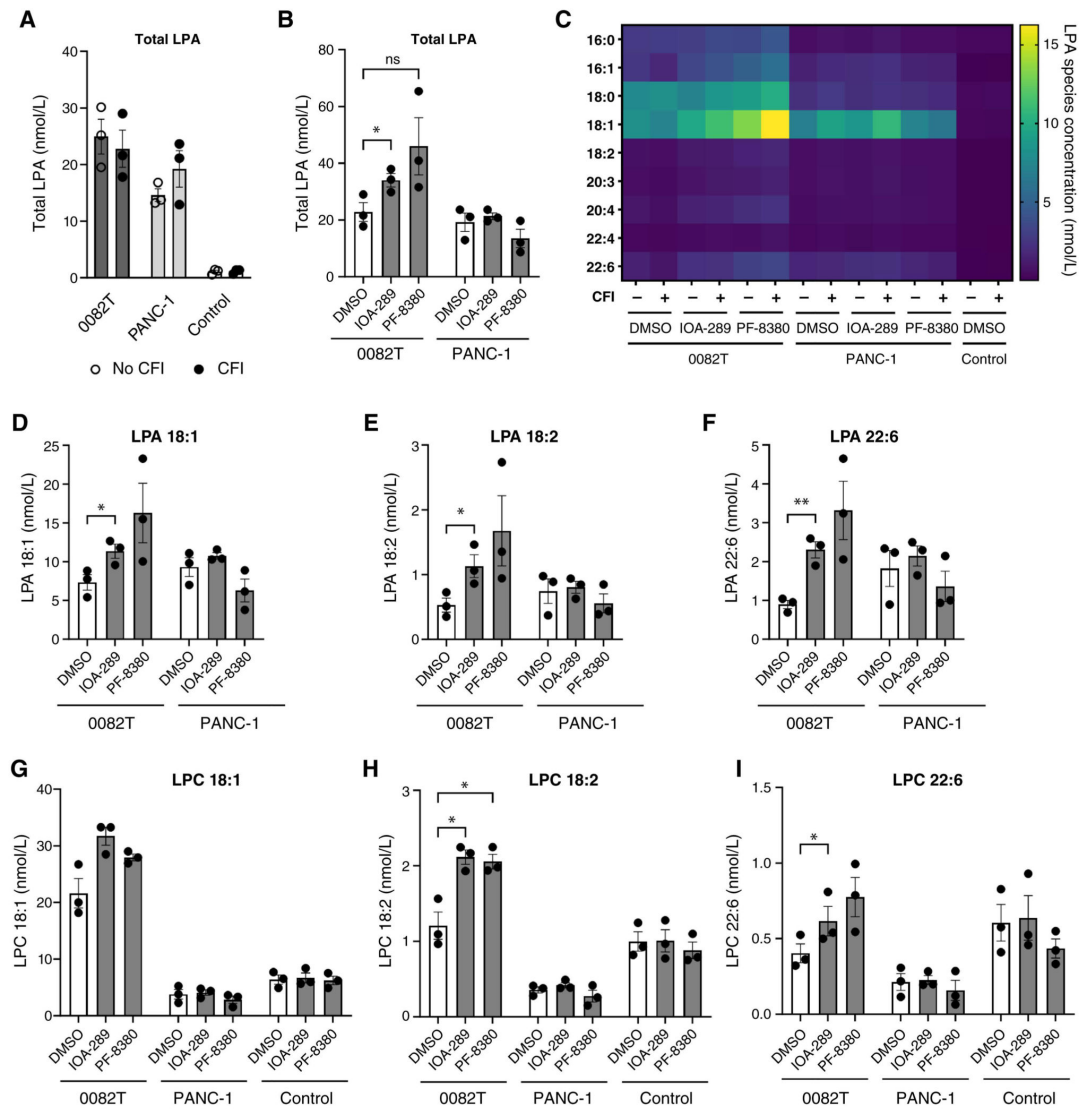
ATX inhibition modulates LPA/LPC levels in conditioned media (CM) of 0082T CAFs. **A**, Total LPA concentration in the CM of 0082T and PANC-1 cells treated with 0.1% DMSO, and control after 48 hours conditioning and with or without CFI (N=3). **B**, Total LPA concentration in the CM of 0082T cells and PANC-1 cells treated with 0.1% DMSO or ATX inhibitors 12 μmol/Lol/L IOA-289 or 12 μmol/Lol/L PF-8380 for 48 hours conditioning with CFI (N=3). Significance determined by two-way ANOVA and post-hoc Dunnett comparisons. ‘ns’ denotes non-significance and ‘*’ indicates *p* value <0.05. **C**, Heatmap showing concentration of individual LPA species in the CM of 0082T and PANC-1 cells, and in control after 48 hours of conditioning, with (+) and without (-) CFI when treated with 0.1% DMSO, 12 μmol/Lol/L IOA-289 or 12 μmol/Lol/L PF-8380 (N=3). **D-E-F**, Concentration of 18:1 LPA (**D**), 18:2 LPA (**E**) and 22:6 LPA (**F**) in the CM of 0082T and PANC-1 cells after treatment with 0.1% DMSO or ATX inhibitors 12 μmol/Lol/L IOA-289 or 12 μmol/Lol/L PF-8380 for 48 hours of conditioning and with CFI (N=3). Significance determined by two-way ANOVA and post-hoc Dunnett comparisons. ‘ns’ denotes non-significance. ‘*’ and ‘**’ indicates p values <0.05, and <0.01 respectively. **G-H-I**, Concentration of 18:1 LPC (**G**), 18:2 LPC (**H**) and 22:6 LPC (**I**) in the CM of 0082T and PANC-1 cells, and in control after treatment with 0.1% DMSO or ATX inhibitors 12 μmol/Lol/L IOA-289 or 12 μmol/Lol/L PF-8380 for 48 hours of conditioning and with CFI (N=3). Significance determined by two-way ANOVA and post-hoc Dunnett comparisons. ‘ns’ denotes non-significance. ‘*’ and ‘**’ indicates *p* value <0.05, and <0.01 respectively.

As LPA is rapidly degraded by cell surface LPPs (LPP1-3) (38), LPA concentration measured in CM may not directly reflect ATX activity. LPP expression profiles in PDAC and 0082T cell lines from CCLE and pancreatic scRNA-seq datasets were consistent in showing that LPP2 is the most expressed in the majority of PDAC cancer cell lines, whereas CAFs predominantly express LPP1 and LPP3 (Figure S6A-D). To investigate the LPA-generating capacity of secreted ATX in CM in the absence of LPPs, we included a 24-hour cell-free incubation (CFI) step. We first explored extracellular LPA levels in 0082T and PANC-1. Although, in our hands, only 0082T cells express ATX, LPA was detectable in CM from 0082T and PANC-1 (Figure 4A). In addition, CFI did not reveal any LPA-generating activity of ATX: total LPA levels were similar in 0082T CM before and after CFI (Figure 4A). These results indicated that LPA, is mainly generated by an ATX-independent pathway, which is difficult to reconcile with our results showing an LPAR-dependent decrease in CTGF after ATX inhibition in the CAF cell line. Treatment of 0082T cells with IOA-289 and PF-8380 confirmed that LPA levels were not decreased in response to ATX inhibition, and contrary to expectations, a significant increase in LPA content was observed with IOA-289 (Figure 4B), especially for the 18:1, 18:2 and 22:6 LPA species (Figure 4C-F). Such an effect was not observed with PANC-1, which do not express ATX (Figure 4C-F). LPC, the main ATX substrate, was only significantly detected in CM from 0082T, and not in PANC-1 CM where the levels were lower than in the control, indicating that the uptake of LPC in these cells is higher than the release (Figure 4G-I). In 0082T CM, LPC species, 18:1 LPC and 18:2 LPC, were also significantly increased in response to IOA-289 (Figure 4G-I). The RNA-seq data, which revealed only four significantly modulated genes in response to IOA-289 (Figure 3C and S5), does not provide evidence of compensatory mechanisms or a profound shift in LPA metabolism in response to ATX inhibition. To explain this, we reasoned that either ATX inhibitors paradoxically increase ATX activity towards specific substrates, or that ATX has another function than LPC hydrolysis in this system.

### ATX loss of function regulates LPA signaling and CTGF secretion in 0082T CAFs

**Figure 5:**
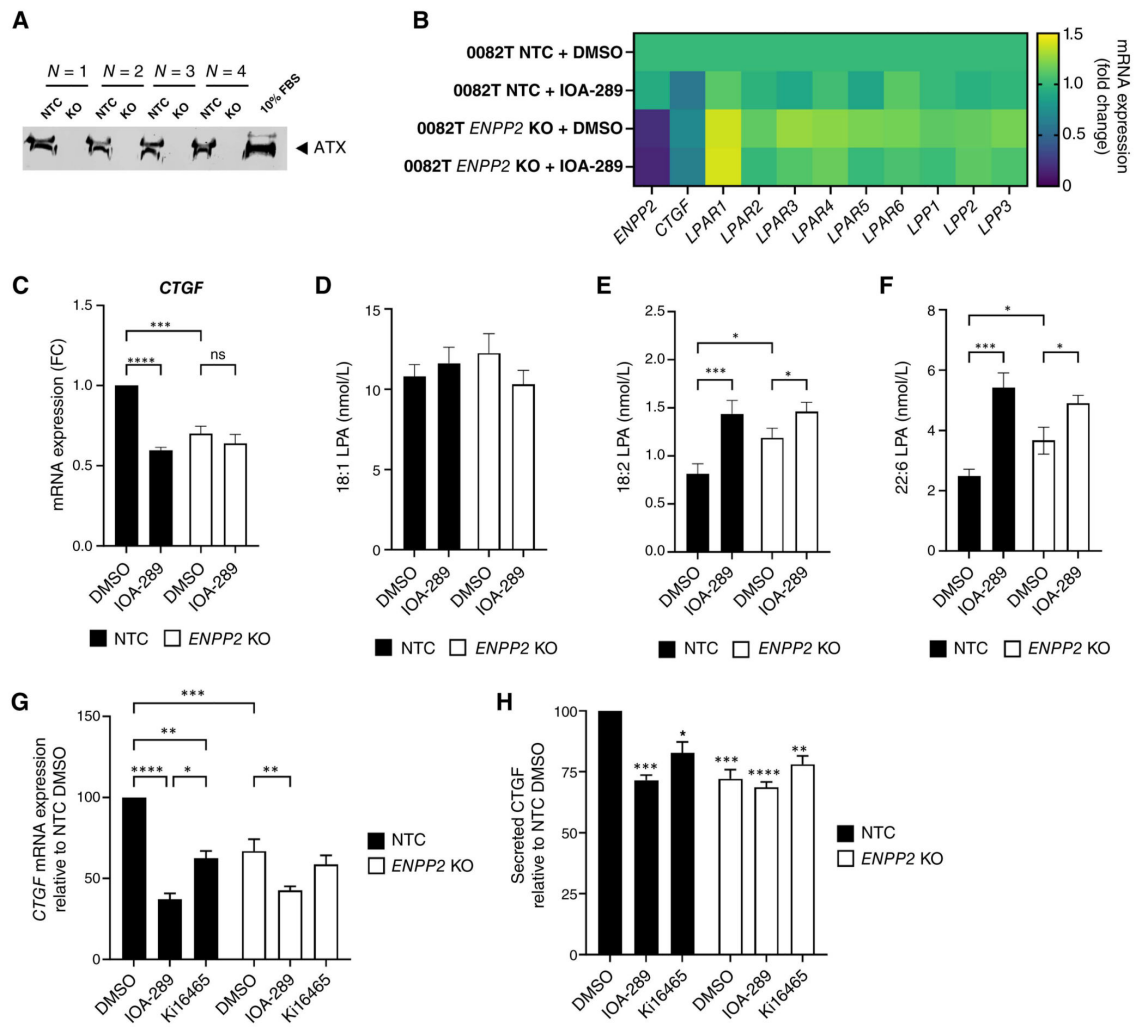
ATX genetic depletion in 0082T CAFs mimics the effects of IOA-289 inhibitor. **A**, Validation of ATX depletion by western blot in CM generated from 4 independent 0082T *ENPP2* KO pools compared to their corresponding 0082T non-targeting controls (NTC). **B**, Gene expression profile in 0082T NTC and 0082T *ENPP2* KO after 48-hour treatment with 0.1% DMSO (vehicle control), 12 μmol/L IOA-289, relative to vehicle control NTC (N=4). *RPL13A* was used as housekeeping gene. **C**, *CTGF* mRNA expression in 0082T NTC and corresponding *ENPP2* KO (N=4) after 48-hour treatment with 0.1% DMSO (vehicle control) or 12 μmol/L IOA-289, relative to vehicle control NTC. *RPL13A* was used as housekeeping gene. Mean ± SEM. Significance determined by a two-way ANOVA and post-hoc Fischer comparisons and ‘***’, and ‘****’ indicates *p* value =0.0001 and <0.0001, respectively. **D-E-F**, Concentration of 18:1 LPA (**D**), 18:2 LPA (**E**) and 22:6 LPA (**F**) in the CM of 0082T NTC and 0082T *ENPP2* KO cells after treatment with 0.1% DMSO or 12 μmol/Lol/L IOA-289 for 48 hours of conditioning and CFI (N=4). Mean ± SEM. Significance determined by a two-way ANOVA, with ‘*’, ‘***’, indicates *p* value <0.05 and <0.0005, respectively. **G-H**, Relative *CTGF* mRNA expression (**G**) and protein secretion (**H**) in NTC 0082T (N=3) and corresponding *ENPP2* KO (N=3 KO using sgRNA-2 and N=2 using sgRNA-1) cells after treatment with 0.1% DMSO (vehicle control), 12 μmol/L IOA-289 or 12 μmol/L Ki16425 for 48 hours. *GAPDH* and *HPRT1* were used as housekeeping genes. Mean ± SEM. Significance determined by a two-way ANOVA and post-hoc Turkey comparisons. ‘*’, ‘**’, ‘***’, and ‘****’ indicates *p* value <0.05, <0.005, <0.0005, and <0.0001 respectively.

To determine whether the increase in LPA content in 0082T CM was due to a stimulated production of LPA by ATX inhibitors, or to an off-target effect of ATX-inhibitors, we performed a genetic depletion of the ATX gene, *ENPP2*, in 0082T cells using CRISPR/Cas9. After validation of ATX depletion in *ENPP2* KO pools (Figure 5A,B), ATX-depleted cells and controls were treated with DMSO or IOA-289. The decrease in CTGF expression and secretion (Figure 5B,C) was associated with an increase in 18:2 LPA and 22:6 LPA species (Figure 5D-F) in control cells treated with ATX inhibitor as previously shown but it was also observed in *ENPP2* KO 0082T cells with and without the inhibitor. Importantly, no changes in expression of LPPs were observed in *ENPP2* KO cells (Figure 5B). These results confirmed that the loss of ATX function is the driver of the CTGF decrease and LPA increase, and that the inhibitor is acting on-target. The increase in LPA species and decrease in CTGF expression in *ENPP2* KO cells was not always as strong as with IOA-289 treatment (Figure 5C-H). This could be due to residual ATX protein in the CRISPR KO pools, or remodeling in the *ENPP2* KO. ATX depletion led to an increase in *LPAR1* expression which was probably a feedback regulation in response to the decrease in ATX/LPA signaling (Figure 5B). Finally, the use of Ki16425 (LPAR1-3 inhibitor) did not fully recapitulate the decrease in CTGF observed in response to ATX loss of function, suggesting that ATX/LPA signaling that led to CTGF reduction may also involve LPAR5-6 receptors (Fig 5G-H).

While the decrease in CTGF expression consecutively to the loss of ATX/LPA signaling and LPAR antagonism suggests a decrease in LPA signalling, the increase in extracellular LPA suggests that ATX has another function than LPC hydrolysis. A recent study highlighted a dual function for ATX, acting both as an LPA-producing enzyme and as a chaperone delivering LPA to its receptors. The accumulation of LPA in 0082T CM could be explained by the loss or inhibition of ATX chaperone function, reducing LPA binding and delivery to its receptor, thereby decreasing LPA internalization or degradation. The increase in LPA could also reflect a metabolic remodeling or a reduced turnover of LPA, in response to the loss of ATX chaperone function and subsequent decreased LPA signaling.

## Discussion

In this study, we show that cells of the TME, rather than tumor cells, are the major source of ATX in PDAC and demonstrate that the tumor-promoting role of pancreatic CAF-derived ATX can be driven by both autocrine and paracrine signaling, and likely involves the previously reported LPA chaperone function. We have, therefore, revealed a novel mechanism by which the selective clinical-stage ATX inhibitor, IOA-289, could restrict tumor progression.

Auciello and colleagues established a model of ATX-regulated PDAC biology in which both CAF and tumor-derived ATX drive tumorigenesis by converting LPC to LPA, which stimulates LPAR-driven PDAC pathogenesis (15). Our findings refine this model, to include regulation of CTGF by ATX via LPA-LPAR signaling, and highlights that CAFs, rather than tumor cells, contribute to the ATX load in PDAC TME. We also describe a putative role for the LPA-chaperone function of ATX and find that its catalytic function is not as important in this *in cellulo* system.

We demonstrated that ATX pharmacological inhibition, or genetic depletion, significantly reduces secretion of the pro-fibrotic factor, CTGF, by 0082T CAF cells, through reduced LPA-mediated LPAR activation. In mouse models of PDAC, FG-3019, an anti-CTGF monoclonal antibody, in combination with gemcitabine, significantly increased intra-tumoral apoptosis and reduced tumor size compared to gemcitabine alone (39,40). Recent *in vivo* data supports our findings showing that treatment of mice bearing orthotopic PDAC tumors with IOA-289 led to a significant decrease in CTGF plasma concentration (10). When combined with gemcitabine, however, IOA-289 treatment had a synergistic effect leading to a more drastic reduction in plasma CTGF and longer overall survival of mice (10). Our data suggest that treatment with ATX inhibitors has the potential to phenocopy CTGF antagonism whilst also modulating other LPA-regulated processes in PDAC.

Our data suggest that other LPA-producing pathways might play an important and complementary role to ATX in the LPA/LPAR biology. A likely candidate for this alternative pathway is membrane associated phospholipase A1 (PLA-1) enzyme. Phospholipases A produce LPA from phosphatidic acid (PA) and are the main source of extracellular LPA besides ATX (41). In support of this, the concentration of LPA does not increase with CFI, suggesting that the enzyme responsible for generating it, or the source of its substrate, is likely cell associated.

IOA-289 reduces the circulating levels of LPA in mouse models and healthy human volunteers (9), indicating that ATX contributes to LPA generation *in vivo*. The plasma level of LPC is around 100-200 μmol/L (42) which is far higher than the level released by 0082T cells in our experiments (100-200 nM). ATX is known to undergo product inhibition, and its activity is therefore sensitive to substrate levels. In cell models, it has been previously shown that addition of LPC increased ATX-mediated signaling (43) indicating that, in our experiments, LPC concentrations may be too low to drive appreciable ATX enzymatic activity and that the level of ATX enzymatic activity is likely to depend on the LPC level in the TME.

Recent studies have revealed that ATX is a dual-function protein that acts as an LPA-producing enzyme and as an LPA chaperone, facilitating delivery of LPA to its receptors and activating LPAR signaling pathways independently of its catalytic activity (44,45). Our paradoxical finding that genetic depletion, or pharmacological inhibition, of ATX results in increased extracellular levels of 18:2 and 22:6 LPA, yet reduced *CTGF* expression downstream of LPAR signaling, led us to conclude that ATX is acting primarily as a chaperone in this system rather than enzymatically. This gives context to previous work in lung fibrosis where ATX is increased in bronchoalveolar lavage fluid (BALF) with bleomycin injury (46). The majority of LPA present in BALF after injury was 22:5 and 22:6, and these species were increased upon ATX inhibition, mirroring what we observed in 0082T CM upon ATX inhibition and depletion. The authors concluded that ATX was not required for LPA production in response to lung injury (46), but our data suggests that ATX was nevertheless potentially involved in LPA signaling through its chaperone function.

ATX has a unique tripartite binding site: a catalytic bimetallic site that accommodates the glycerol moiety of lipid substrates, a substrate-binding (orthosteric) hydrophobic pocket that binds lipid acyl chains, and an allosteric tunnel that can accommodate various steroids and LPA and which has been proposed to serve as an “exit channel”(43). The ATX LPA chaperone function is not limited to the tunnel-bound LPA but extends to the orthosteric site (LPC/LPA binding pocket) and regulates selective cellular responses independently of catalytic activity (45). In the work described here, we observed differences in responses to ATX inhibitors. This may be explained by their different modes of action: PF-8380 occupies the catalytic site and the hydrophobic pocket whereas IOA-289 instead blocks the hydrophobic binding pocket and the allosteric LPA binding site (43). The functional significance of the different LPA species according to their binding to ATX is also a key question and further work is required to determine how 18:2, and if 22:6, LPA-bound ATX activates LPAR signaling and whether they stimulate the same signaling pathways in PDAC TME cells.

LPAR expression is heterogeneous across cell types within PDAC tumors, therefore inhibiting ATX could further impact cells of the PDAC TME. For example, signaling via LPAR6 inhibits CD8+ T cell migration (44) and LPA inhibits CD8+ T cell activation via LPAR5 (47). ATX secreted by melanoma cells functions as an LPA-producing chaperone which is chemorepulsive for CD8+ T cells and its inhibition by IOA-289 restores T cell migration (44). In non-small cell lung cancer, ATX suppresses cytotoxic T cells via LPAR5 to promote anti-PD1 resistance (48). In addition, IOA-289 treatment increased the recruitment of tumor-infiltrating CD8+ T cells in orthotopic mouse models of pancreatic cancer treated with gemcitabine (10). Interestingly, recent work by Wu and colleagues showed that PDAC cells upregulated LPAR4 in response to chemotherapy-induced stress (49), indicating that LPA-LPAR signaling may be more dynamic than previously appreciated.

Additional *ENPP2*-expressing cells were identified in the PDAC TME population, including endothelial cells and macrophages. Tumor-associated macrophages (TAMs) have been associated with poor prognosis, and pre-clinical studies have demonstrated a multi-faceted role of TAMs in promoting PDAC (50). More in-depth mechanistic studies are required to decipher the ATX-related autocrine and paracrine signaling that occurs in PDAC, and their contribution to cancer cell growth and immunosuppressive mechanisms.

Unlike PDAC, in cancers such as hepatocellular carcinoma and kidney renal clear cell carcinoma both tumor and TME cells contribute to the increase in *ENPP2* expression. This highlights the complexity of ATX biology that varies between cancer indications. From a more general perspective, our findings open new research avenues into the dual function of ATX according to the environmental context, considering both the species and the levels of LPA and LPC, and the LPAR expression profiles of the target cells.

Overall, the results of this study not only complement and refine previous evidence describing the important role of ATX in promoting CAF-induced pro-tumorigenic signaling in PDAC, but also further highlight the importance of ATX LPA chaperone function in LPA-LPAR signaling. This will help to interpret future research and clinical data from clinical trials evaluating ATX inhibitors.

## Supplementary Material

FS1

FS2

FS3

FS4

FS5

FS6

Supplementary materials & methods

## Data Availability

Data associated with this study are available in the article and its Supplementary Data. RNA-seq data have been deposited in the ArrayExpress database (www.ebi.ac.uk/arrayexpress) at the European Bioinformatics Institute of the European Molecular Biology Laboratory (EMBL-EBI) under accession number E-MTAB-14494.
