# Supplementary material for: Pancreatic CAF-derived Autotaxin (ATX) drives autocrine CTGF expression to modulate pro-tumorigenic signaling
Source: Mol Cancer Ther. Author manuscript; Available in PMC 2025 Oct 23. (PMC7618285; doi:10.1158/1535-7163.MCT-23-0522)
Supplement: FS1 [file EMS208572-supplement-FS1.docx]

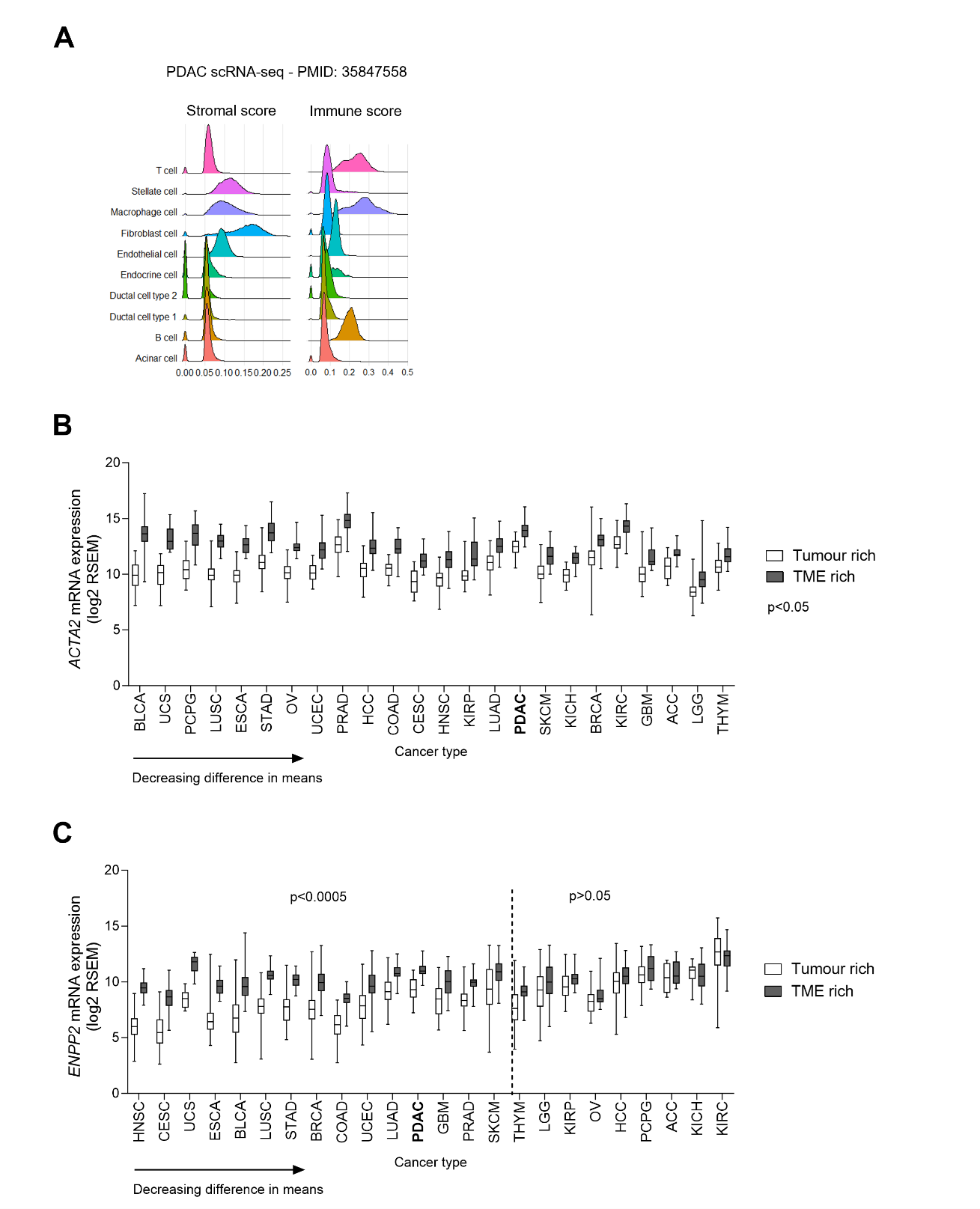


**Figure S1. Supplemental to Figure 1.**

**A**, Validation of ESTIMATE stromal and immune gene signatures with the scRNA-seq dataset from (Chijimatsu R, 2022).

**B-C**, *ACTA2 (****B****)* and *ENPP2 (****C****)* mRNA expression in tumour-rich and TME-rich samples across several cancers in the TCGA PanCancer Atlas study. Groupings were defined used ESTIMATE scores. Significance determined by two-way ANOVA. RSEM: RNA-Seq by Expectation-Maximization.

Cancer abbreviations are as follows: ACC- Adrenocortical Carcinoma; BLCA-Bladder Cancer; BRCA-Breast Invasive carcinoma; CESC-Cervical squamous cell carcinoma and endocervical adenocarcinoma; COAD-Colon adenocarcinoma; ESCA-Esophageal carcinoma; GBM-Glioblastoma multiforme; HCC-Hepatocellular Carcinoma; HNSC-Head and Neck squamous cell carcinoma; KICH-Kidney Chromophobe; KIRC-Kidney renal clear cell carcinoma; KIRP-Kidney renal papillary cell carcinoma; LGG: Brain Lower Grade Glioma; LUAD-Lung adenocarcinoma; LUSC-Lung squamous cell carcinoma; OV-Ovarian serous cystadenocarcinoma; PDAC-Pancreatic ductal adenocarcinoma; PCPG-Pheochromocytoma and Paraganglioma; PRAD-Prostate adenocarcinoma; SKCM-Skin Cutaneous Melanoma; STAD: Stomach adenocarcinoma; THCA-Thyroid carcinoma; Uterine Cancer; THYM: Thymoma ; UCS-Uterine Carcinosarcoma; UCEC-Uterine Corpus Endometrial Carcinoma.
