# Supplementary material for: Pancreatic CAF-derived Autotaxin (ATX) drives autocrine CTGF expression to modulate pro-tumorigenic signaling
Source: Mol Cancer Ther. Author manuscript; Available in PMC 2025 Oct 23. (PMC7618285; doi:10.1158/1535-7163.MCT-23-0522)
Supplement: FS2 [file EMS208572-supplement-FS2.docx]

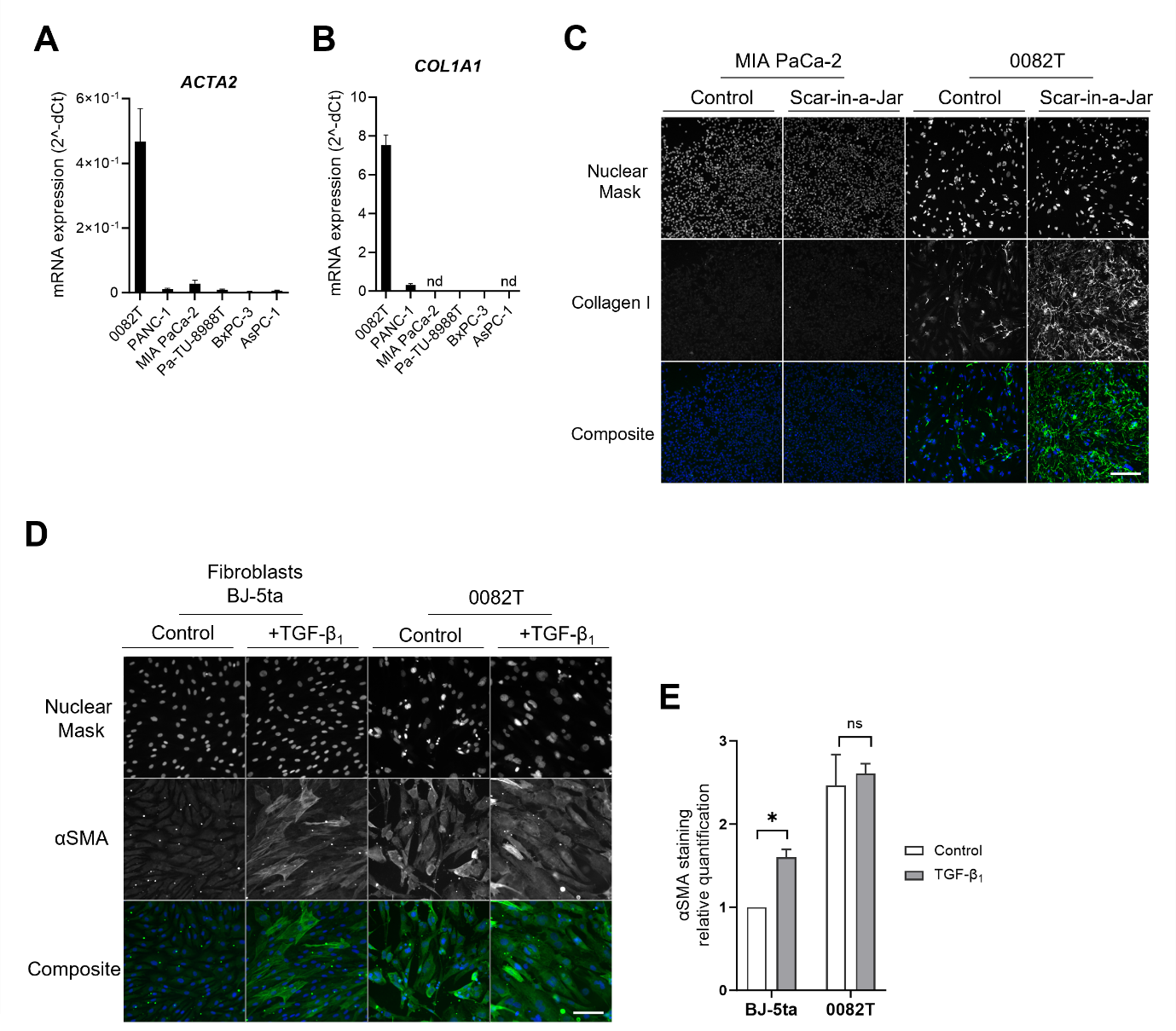


**Figure S2. Validation of 0082T CAF phenotype.**

**A-B,***mRNA*expression of *ACTA2* (**A**) and *COL1A1* (**B**) in 0082T CAFs and PDAC cell lines cultured in SF DMEM for 48 hours (N=3). *RPL13A* was used as housekeeping gene. **C**, Representative images of extracellular Type I collagen deposition by MIA PaCa-2 and 0082T cells in normal and scar-in-a-jar culture condition (N=3). Scale bar = 250 µM. **D-E**, αSMA immunofluorescence of fibroblasts BJ-5ta and pancreatic 0082T cells after 72-hour treatment ± 2 ng/ml TGF-β1. Representative images (**D**) and quantification (**E**) of ɑSMA expression of N=3 independent biological repeats. Paired *t*-test shows a *p* value <0.05 (*) and non-significance is denoted by ‘ns’. Scale bar = 250 µM.
