# Supplementary material for: Pancreatic CAF-derived Autotaxin (ATX) drives autocrine CTGF expression to modulate pro-tumorigenic signaling
Source: Mol Cancer Ther. Author manuscript; Available in PMC 2025 Oct 23. (PMC7618285; doi:10.1158/1535-7163.MCT-23-0522)
Supplement: FS3 [file EMS208572-supplement-FS3.docx]

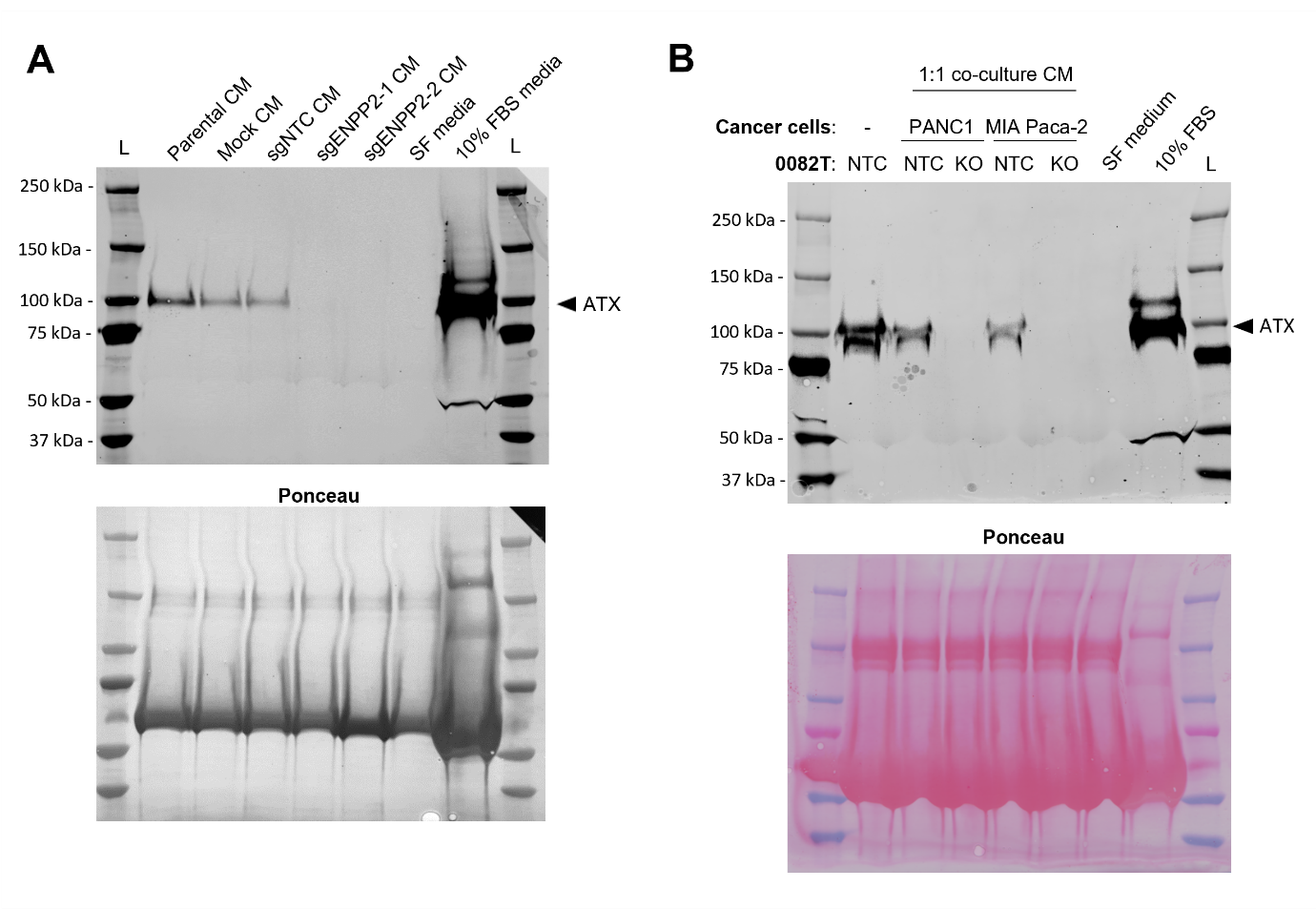


**Figure S3. ATX detection by western blots. A**, ATX antibody validation using CM generated from the parental CAF cell line 0082T, mock and NTC controls of 0082T CRISPR KO experiment, and *ENPP2* KO 0082T generated with 2 different guides (sgENPP2-1 and sgENPP2-2). SF medium (Serum free DMEM + 0.5% BSA) and 10% fetal bovine serum (FBS) DMEM are used as controls. Ponceau staining is shown as a loading control. Representative western blot of at least N=3 CRISPR KO experiments. **B**, ATX detection in CM generated from co-culture of CAF 0082T (NTC or *ENPP2* KO) with PDAC cell lines (PANC-1 or MIA PaCa-2) at a 1:1 ratio. SF medium and 10% FBS DMEM are used as controls. Representative western blot of N=3 experiments performed with 3 independent 0082T *ENPP2* KO pools. Ponceau staining is shown as a loading control.

NTC, Non-targeting control. KO, *ENPP2* knockout. L, Ladder.
