# Supplementary material for: Pancreatic CAF-derived Autotaxin (ATX) drives autocrine CTGF expression to modulate pro-tumorigenic signaling
Source: Mol Cancer Ther. Author manuscript; Available in PMC 2025 Oct 23. (PMC7618285; doi:10.1158/1535-7163.MCT-23-0522)
Supplement: FS4 [file EMS208572-supplement-FS4.docx]

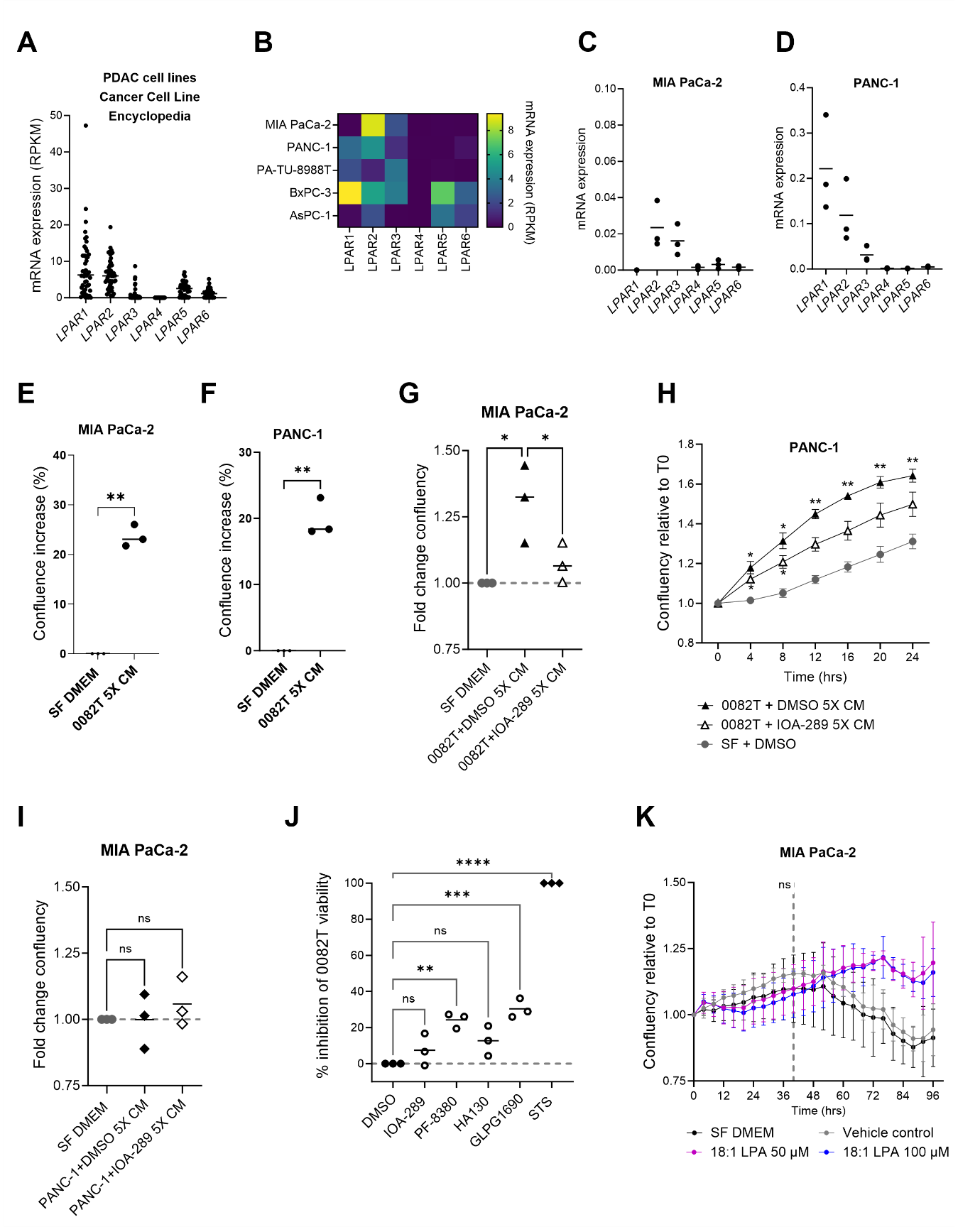


**Figure S4. LPAR expression in PDAC cell lines and ATX-mediated pro-tumorigenic stimulation by 0082T conditioned media.**

**A-B**, *LPAR1-6* mRNA expression in PDAC cancer cell lines from the Cancer Cell Line Encyclopedia (CCLE) (RNAseq - Reads Per Kilobase of transcript per Million mapped reads or RPKM). (**A**) Expression in all PDAC cancer cell lines (n=46) with line at median. (**B**) Heatmap of expression profile in MIA PaCa-2, PANC-1, PA-TU-8988T, BxPC-3 and AsPC-1. **C-D**, LPA receptors expression in 48-hour serum starved MIA PaCa-2 cells (**C**) and PANC-1 cells (**D**) (N=3). **E**, Percentage of confluence increase after 40 hours of MIA PaCa-2 cells treated with concentrated 0082T CM (5X concentration – generated in serum free (SF) DMEM for 48 hours) compared to cells grown in SF DMEM (N=3). Paired *t*-test shows a *p* value <0.005 indicated by ‘**’. **F,**Percentage of confluence increase after 24 hours of PANC-1 cells treated with 5X concentrated 0082T CM (generated for 48 hours) compared to cells grown in SF DMEM (N=3). Paired *t*-test shows a *p* value <0.01 indicated by ‘**’. **G,** MIA PaCa-2 growth increase relative to SF DMEM at 40 hours upon treatment with the indicated 5X CAF CM (N=3). 5X CM is generated from 0082T treated with 0.1% DMSO or 12 μmol/L IOA-289 in SF DMEM for 72 hours. Ordinary one-way ANOVA shows a *p* value <0.05 indicated by ‘*’. **H**, Relative PANC-1 growth over time in hours (hrs) upon treatment with SF DMEM + 0.1% DMSO, or the indicated 5X 0082T CM (N=3). 5X CM is generated from 0082T treated with 0.1% DMSO (0082T + DMSO 5X CM) or 12 μmol/L IOA-289 (0082T + IOA-289 5X CM) in SF DMEM for 48 hours. Data represents means -/+ SEM of N=3. ‘*’ and ‘**’ indicate a *p* value <0.05 and <0.005 respectively compared to the SF+DMSO condition, based on two-way ANOVA and post-hoc Turkey comparisons. **I,** MIA PaCa-2 growth increase relative to SF DMEM at 40 hours upon treatment with the indicated 5X PDAC CM (N=3). 5X CM (supplemented with 0.1% DMSO) is generated from PANC-1 treated with 0.1% DMSO or 12 μM IOA-289 in SF DMEM for 72 hours. The *p* value ≥0.05 is indicated by ‘ns’ for non-significance based on ordinary one-way ANOVA.  **J**, Inhibition (%) of 0082T cell viability assessed by CellTiter-Glo assays at 48 hours post-treatment with 0.1% DMSO, 1 µM Staurosporine/STS (cell death control) or 12 µmol/L ATX inhibitors (IOA-289, PF8380, HA130 or GLPG1690). Significance determined by two-way ANOVA and post-hoc Turkey comparisons. ‘ns’ denotes non-significance. ‘**’, ‘***’, ‘****’ indicate *p* value <0.005, <0.0005 and <0.0001 respectively. **K**, MIA PaCa-2 growth relative to SF DMEM over 4 days upon treatment with SF DMEM, vehicle control for 18:1 LPA, 50 μmol/L 18:1 LPA, or 100μmol/L 18:1 LPA (N=3). Grey dashed line indicates the 40-hour timepoint. Significance determined by one-way ANOVA with Tukey’s multiple comparisons test for any pair of data at 40 hours.
