# Supplementary material for: Pancreatic CAF-derived Autotaxin (ATX) drives autocrine CTGF expression to modulate pro-tumorigenic signaling
Source: Mol Cancer Ther. Author manuscript; Available in PMC 2025 Oct 23. (PMC7618285; doi:10.1158/1535-7163.MCT-23-0522)
Supplement: FS5 [file EMS208572-supplement-FS5.docx]

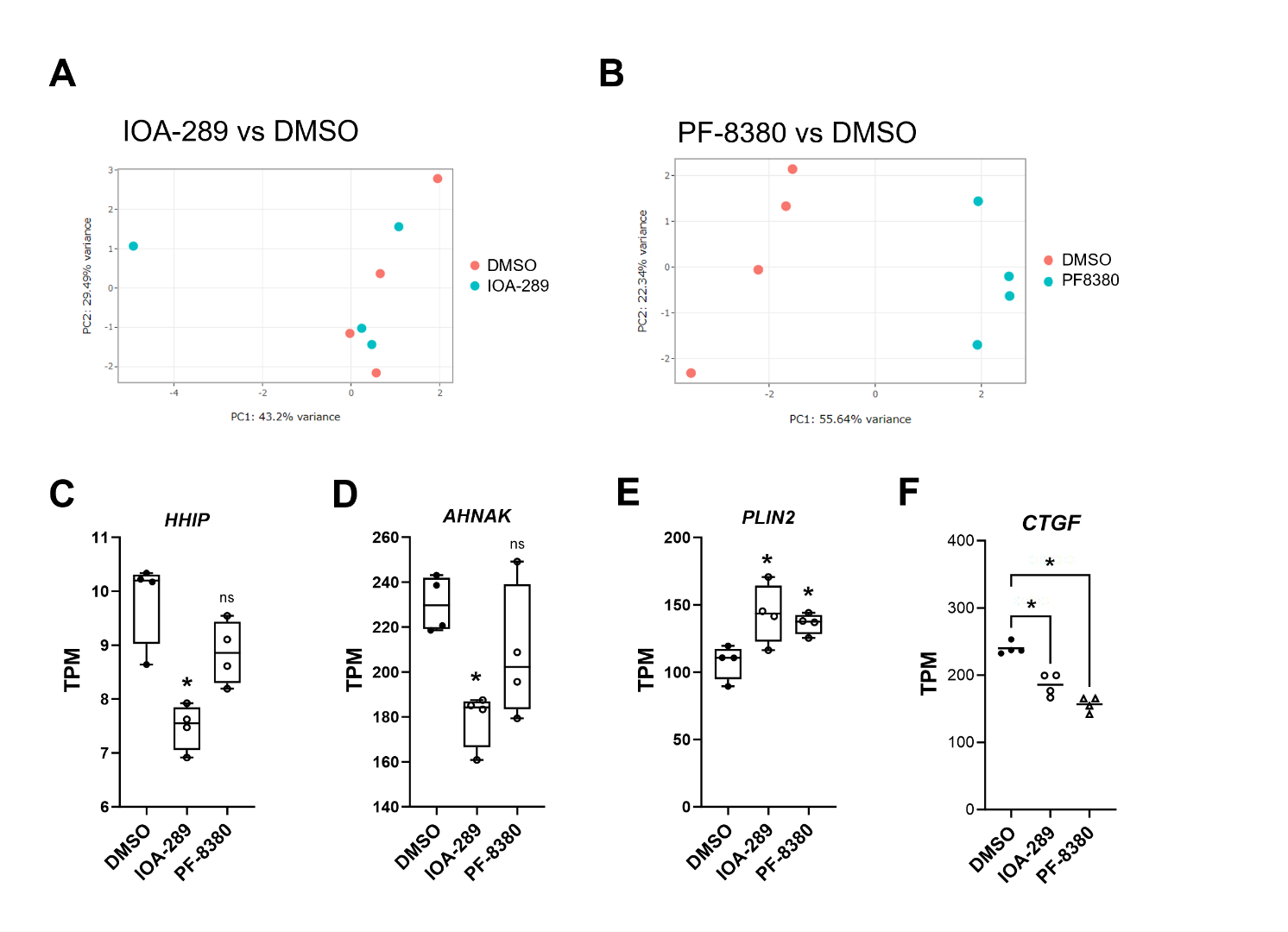


**Figure S5. 0082T CAFs RNA-seq data after treatment with ATX inhibitors.**

**A-B,**Principal component analysis (PCA) of 24-hour treatment of 0082T cells with 0.1% DMSO vs 12 μmol/L IOA-289 (**A**) or 0.1% DMSO vs 12 μmol/L PF-8380 (**B**) in serum free DMEM (N=4). **C-D-E-F**, Expression in transcript per million (TPM) in 0082T cells treated with 0.1% DMSO, 12 μmol/L IOA-289 or 12 μmol/L PF-8380 for 24 hours of *HHIP* (**C**), *AHNAK* (**D**) *PLIN2* (**E**) and *CTGF* (**F**) (N=4). Adjusted *p* values are calculated by Wald test shown. ‘ns’ denotes non-significance and ‘*’ indicates a *p* value <0.0
