# Supplementary material for: Pancreatic CAF-derived Autotaxin (ATX) drives autocrine CTGF expression to modulate pro-tumorigenic signaling
Source: Mol Cancer Ther. Author manuscript; Available in PMC 2025 Oct 23. (PMC7618285; doi:10.1158/1535-7163.MCT-23-0522)
Supplement: FS6 [file EMS208572-supplement-FS6.docx]

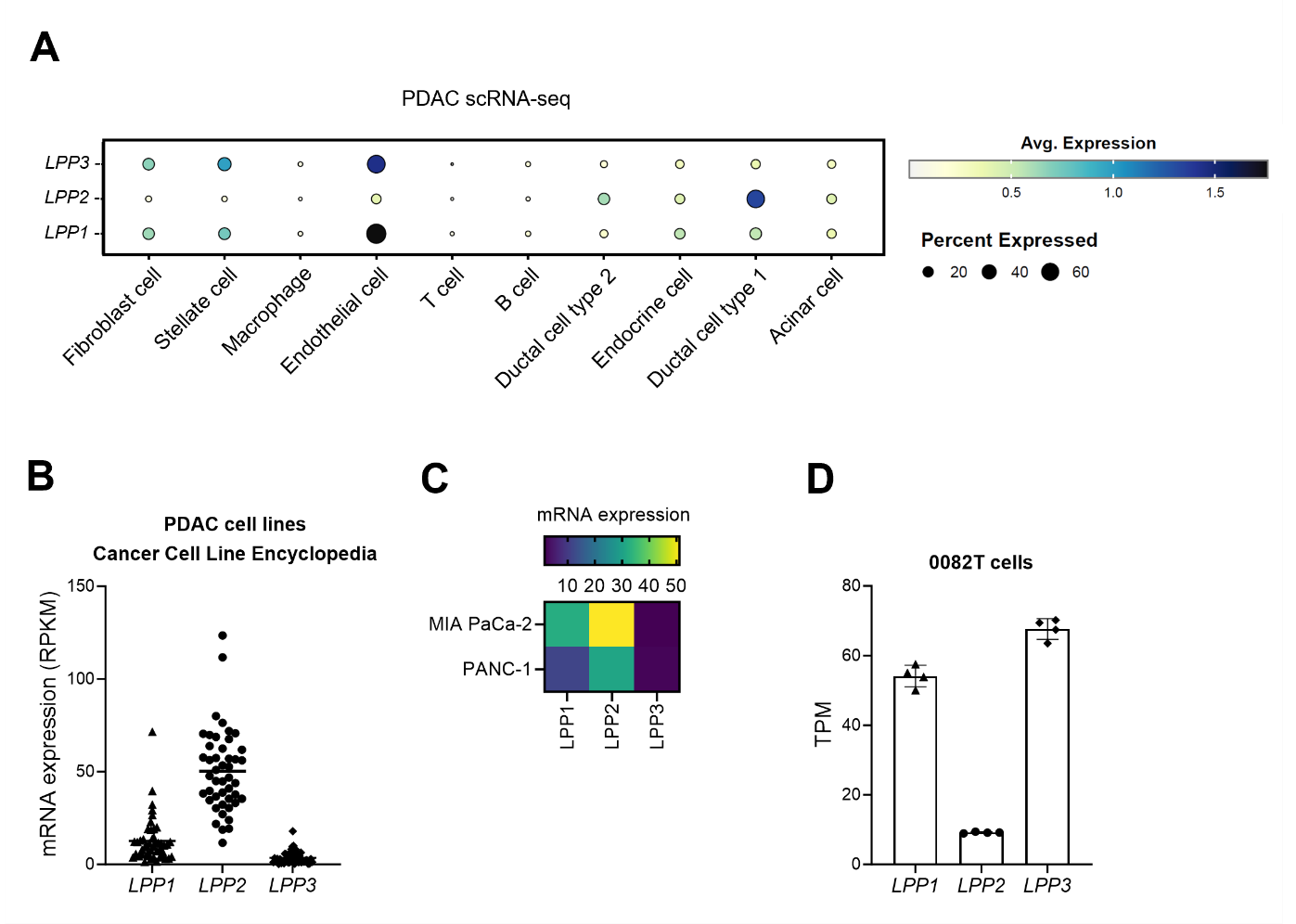


**Figure S6. LLPs expression in cancer and CAFs cells from PDAC. A**, *LPP1-3 and PLA1A* expression in cells of TME and tumour cells in the pancreatic scRNA-seq dataset from (Chijimatsu R, 2022). **B**, *LPP1-3* mRNA expression (RNA-seq RPKM) in all PDAC cancer cell lines in CCLE (n=46), showing mean and all individual values. **C**, Heatmap of mean *LPP1-3* mRNA expression (RNA-seq RPKM) in MIA PaCa-2 and PANC-1 cells. **D**, *LPP1-3* mRNA expression in transcript per million (TPM) in 0082T cells treated with DMSO in serum free media with 0.5% FAF BSA for 24 hours (N=4).
