# Supplementary material for: Pancreatic CAF-derived Autotaxin (ATX) drives autocrine CTGF expression to modulate pro-tumorigenic signaling
Source: Mol Cancer Ther. Author manuscript; Available in PMC 2025 Oct 23. (PMC7618285; doi:10.1158/1535-7163.MCT-23-0522)
Supplement: Supplementary materials & methods [file EMS208572-supplement-Supplementary_materials___methods.docx]

**Supplementary Materials and Methods**

- **Compounds synthesis**
- **PF-8380**

Overall Scheme for synthesis of PF-8380:

**Step 1 – Preparation of 1-(tert-butyl) 4-(3,5-dichlorobenzyl) piperazine-1,4-dicarboxylate.**

To (3,5-dichlorophenyl)methanol (3.8 g, 21.475 mmol) in DCM (dichloromethane) (40 mL) was added 1,1’-carbonyldiimdazole (3.83g, 23.623 mmol) and the mixture stirred for 2 h. Tert-butyl piperazine-1-carboxylate (4.0 g, 21.475 mmol) was added and the mixture stirred for a further 2 h. Additional tert-butyl piperazine-1-carboxylate (4.295 mmol, 0.8 g) was added and the mixture stirred for a further 1 h. Water was added and the mixture extracted with DCM. The organic extracts were washed with saturated sodium hydrogen carbonate (Aq.) then brine and then passed through a phase separation cartridge and concentrated. The resultant solid was purified by flash chromatography on SiO_2_ (0-50% ethyl acetate in cyclohexane) to give 1-(tert-butyl) 4-(3,5-dichlorobenzyl) piperazine-1,4-dicarboxylate (7.857 g, 20.18 mmol, 94%)

NMR (1H, 500 MHz, DMSO-*d*6) 7.56 (t 1H), 7.42 (d 2H), 5.07 (s 2H), 3.31 (s 8H), 1.39 (s 9H)

HPLC-MS (C18, formic acid modifier) [M+H]^+^ = 289.13/291.15, Purity 100%

**Step 2 – Preparation of 3,5-dichlorobenzyl piperazine-1-carboxylate**

1-(tert-butyl) 4-(3,5-dichlorobenzyl) piperazine-1,4-dicarboxylate (7.857g, 20.183 mmol) was dissolved in 1.25 M HCl in isopropanol (250 mL) and DCM (25 mL) and stirred for 16 h and then evaporated. The resultant solid was dissolved in methanol (with heating) and loaded onto a strong cation exchange cartridge and washed with methanol. Elution with 2M methanolic ammonia and evaporation of the volatiles gave a gum (5.885 g). The gum was dissolved in DCM and washed twice with brine and the organics evaporated to give 3,5-dichlorobenzyl piperazine-1-carboxylate (5.539g, 19.15 mmol, 95%) as a colourless solid.

NMR (1H, 500 MHz, DMSO-*d*6) 7.55 (t 1H), 7.40 (d 2H), 5.05 (s 2H), 3.30 (br d 4H), 2.63 (m 4H)

HPLC-MS (C18, formic acid modifier) [M+H]^+^ = 289.13/291.15, Purity 100%

**Step A – Preparation of 6-(3-chloropropanoyl)benzo[d]oxazol-2(3H)-one**


DMF (1.5 mL) was added dropwise to aluminium trichloride (9.3 g, 69.92 mmol) at 0 °C (Care- exotherm!) and then the mixture allowed to stir at room temperature for 5 min. Benzo[d]oxazol-2(3H)-one (2.7 g, 20 mmol) was added and the mixture stirred for 10 min. The mixture was cooled to 0 °C and 3-chloropropionyl chloride (2.86 mL, 3.80 g, 30 mmol) was added dropwise and then the mixture heated to 75 °C for 2 h.

After cooling, the mixture was diluted with DCM (5mL) and ice (20 g) was added. The remaining solids were removed by filtrate and triturated with iso-propanol to give 6-(3-chloropropanoyl)benzo[d]oxazol-2(3H)-one (3.28 g, 14.58 mmol, 73%) as a beige solid.

HPLC-MS and NMR data in accordance with literature (1).

**Step 3 Preparation of 3,5-dichlorobenzyl 4-(3-oxo-3-(2-oxo-2,3-dihydrobenzo[d]oxazol-6-yl)propyl)piperazine-1-carboxylate**

3,5-dichlorobenzyl piperazine-1-carboxylate (5.121 g, 17.71 mmol) and 6-(3-chloropropanoyl)benzo[d]oxazol-2(3H)-one (3.805 g, 16.87 mmol, were dissolved in DCM ( 250 mL) and triethylamine (7.0 mL, 5.11 g, 50.60 mmol) and stirred for 16 h.

Water was added and the layers separated. The aqueous was extracted twice with DCM and the combined organics passed through a phase separation cartridge and concentrated. The resultant brown gum was purified by flash chromatography on SiO_2_ (0-5% methanol in DCM over 15 CV). The result brown gum was triturated with diethyl ether to give 3,5-dichlorobenzyl 4-(3-oxo-3-(2-oxo-2,3-dihydrobenzo[d]oxazol-6-yl)propyl)piperazine-1-carboxylate as an off-white solid (6.44 g, 13.496 mmol, 80%)

HPLC-MS (C18, formic acid modifier) [M+H]^+^ = 478.31, Purity: 97%.

NMR (1H, 500 MHz, DMSO-*d*6) 12.00 (s 1H), 7.85 (s 1H), 7.83 (dd 1H), 7.56 (t 1H),

7.41 (d 2H), 7.15 (d 1H), 5.06 (s 2H), 3.38 (m 4H,), 3.17 (t 2H), 2.68 (t 2H), 2.40 (t 4H)

**Step 4 Preparation of 3,5-dichlorobenzyl 4-(3-oxo-3-(2-oxo-2,3-dihydrobenzo[d]oxazol-6-yl)propyl)piperazine-1-carboxylate hydrochloride**

3,5-dichlorobenzyl 4-(3-oxo-3-(2-oxo-2,3-dihydrobenzo[d]oxazol-6-yl)propyl)piperazine-1-carboxylate (16.088g, 33.633mmol) was stirred in DCM (200mL) and treated with 1.25 M HCl in isopropanol (100.90 mmol, 80.7 mL) for 3 h and then evaporated. The solid was triturated with isopropanol to give 3,5-dichlorobenzyl 4-(3-oxo-3-(2-oxo-2,3-dihydrobenzo[d]oxazol-6-yl)propyl)piperazine-1-carboxylate hydrochloride (16.657g, 32.356 mmol, 96%)

HPLC-MS (C18, formic acid modifier) [M+H]^+^ = 478.32, Purity: 98%.

NMR (1H, 500 MHz, DMSO-*d*6) 12.19 (s 1H), 10.84 (s 1H), 7.88 (m 2H), 7.58 (m 1H), 7.46 (m 2H), 7.23 (d 1H), 5.11 (s 2H), 4.10 (br s 2H), 3.62-3.44 (m 8H), 3.09 (br s 2H).

- **IOA-289 (CRT0273750)**

IOA-289 (CRT0273750) was synthesized using the same route detailed in reference (2), however reaction conditions were modified as detailed in the following experimental procedures.

**Overall Scheme for synthesis of IOA-289**

**Step 1 – Preparation of 3-nitro-N-(4-(trifluoromethoxy)benzyl)pyridin-2-amine**

To a suspension of 2-chloro-3-nitropyridine (114 g, 0.719 mol) and (4-(trifluoromethoxy)phenyl)methanamine (132 mL, 165 g, 0.862 mol) in dioxane (1.14 L) was added caesium carbonate (305 g, 0.935 mol) and the mixture heated to 90 °C for 3 h. Water (1.0 L) and brine (0.5 L) were added, then ethyl acetate (1.0 L) and the mixture stirred for 10 min at ambient temperature. The phases were separated, and the aqueous layer extracted with ethyl acetate (1.0 L). The combined organic layers were dried over magnesium sulfate and concentrated to give 3-nitro-N-(4-(trifluoromethoxy)benzyl)pyridin-2-amine (274 g, quantitative yield).

1H NMR (300 MHz, CDCl3) 8.53 (1H, br s), 8.46-8.39 (2H, m), 7.39 (2H, d), 7.19 (2H, d), 6.71 (1H, dd), 4.86 (2H, d) with some residual dioxane also present

UPLC-MS (C18, basic modifier) [M+H]^+^ = 314, Purity = 94.62%

**Step 2 – Preparation of N2-(4-(trifluoromethoxy)benzyl)pyridine-2,3-diamine**

A mixture of 3-nitro-N-(4-(trifluoromethoxy)benzyl)pyridin-2-amine (261 g, 0.83 mol) and iron (116 g, 2.08 mol) in industrial methylated spirits (2.5 L) was heated to 75 °C and slowly treated with concentrated hydrochloric acid (442 mL, 5.00 mol). This caused the reaction to exotherm to reflux, thus external heating was removed and the reflux maintained by the rate of addition. Once addition was complete, the mixture was allowed to cool, and the supernatant decanted from the residual iron and concentrated to remove industrial methylated spirits (~ 2.0 L). The residue was filtered, and the filtrate extracted with dichloromethane (~ 2.0 L). The extract was washed with water (1.0 L) and filtered through a pad of Celite, which was washed with dichloromethane (0.5 L). The combined filtrate was washed with sodium hydroxide solution (2 M, 1.0 L) and then with brine (1.0 L). After drying over magnesium sulfate and concentrating to half volume, heptanes (1.0 L) were added and the resultant solid collected by filtration and dried at 40 °C overnight to give N2-(4-(trifluoromethoxy)benzyl)pyridine-2,3-diamine (182 g, 64% yield). (NB further material was present in the filtrate but was not pursued).

1H NMR (300 MHz, CDCl3) 7.78 (1H, d), 7.41 (2H, d), 7.17 (2H, d), 6.88 (1H, dd), 6.58 (1H, dd)4.64 (2H, dd), 4.43 (1H, br s), 3.19 (2H, br s).

UPLC-MS (C18, basic modifier) [M+H]^+^ = 284, Purity = 97.94%

**Step 3 – Preparation of 3-(3-(4-(trifluoromethoxy)benzyl)-3H-imidazo[4,5-b]pyridin-2-yl)propanoic acid**

To a solution of N2-(4-(trifluoromethoxy)benzyl)pyridine-2,3-diamine (94.72 g, 0.334 mol) in dioxane (0.5 L) was added succinic anhydride (33.46 g, 0.334 mol) and the mixture heated to 85 °C for 4 h. Acetic acid (200 mL) was then added, the temperature was raised to 105 °C, stirred for 48 h and then allowed to cool to ambient temperature. The dioxane was removed in vacuo and the residue azeotroped with toluene (2 x 0.5 L). The resultant solid was triturated with tert-butyl methyl ether (0.5 L) at 60 °C, allowed to cool to ambient temperature and the solids collected by filtration, washed with heptanes (0.25 L) and dried at 40 °C under vacuum. The filtrate was concentrated and the residue recrystallised from isopropyl alcohol (0.25 L), collected by filtration, washed with heptanes (0.25 L) and dried at 40 °C under vacuum. The two batches were combined to give 3-(3-(4-(trifluoromethoxy)benzyl)-3H-imidazo[4,5-b]pyridin-2-yl)propanoic acid (103.21 g, 84% yield).

1H NMR (300 MHz, CDCl3) 8.36 (1H, dd), 8.03 (1H, dd), 7.26-7.13 (5H, m), 5.52 (2H, s), 3.12 (2H, dd), 2.99 (2H, dd)

UPLC-MS (C18, basic modifier) [M+H]^+^ = 365.97, Purity = 100%

**Step 4 – Preparation of (S)-N-(1-(4-chlorophenyl)ethyl)-3-(3-(4-(trifluoromethoxy)benzyl)-3H-imidazo[4,5-b]pyridin-2-yl)propanamide**

A mixture of 3-(3-(4-(trifluoromethoxy)benzyl)-3H-imidazo[4,5-b]pyridin-2-yl)propanoic acid (103 g, 0.282 mol), (S)-1-(4-chlorophenyl)ethanamine (46.1 g, 0.296 mol) and Hunig’s base (93 mL, 0.564 mol) in ethyl acetate (1.0 L) was cooled to 0 °C. A solution of T3P (propylphosphonic anhydride; 50 wt% in ethyl acetate, 254 mL, 0.423 mol) was then added over 1 h, maintaining the temperature below 20 °C. After stirring at ambient temperature for 18 h, sodium hydroxide solution (aqueous, 2 M, 0.5 L) was added and the mixture stirred for 10 min. The phases were separated, and the aqueous layer extracted with ethyl acetate (0.5 L). The combined organic layers were washed with brine (1 L), dried over magnesium sulfate and concentrated. The resultant solid was purified by dry-flash chromatography on SiO_2_ (80-100% ethyl acetate in heptanes). Product containing fractions were combined and concentrated. The resultant solid was triturated in 1:1 ethyl acetate/heptanes, collected by filtration and dried at 40 °C under vacuum to give (S)-N-(1-(4-chlorophenyl)ethyl)-3-(3-(4-(trifluoromethoxy)benzyl)-3H-imidazo[4,5-b]pyridin-2-yl)propanamide (102 g, 72% yield).

1H NMR (300 MHz, CDCl3) 8.38 (1H, dd), 7.95 (1H, dd), 7.28-7.06 (9H, m), 6.48 (1H, br d), 5.48 (2H, q), 4.98 (1H, quint), 3.18-3.01 (2H, m), 2.91-2.76 (2H, m), 1.38 (3H, d)

UPLC-MS (C8, basic modifier) [35M+H]^+^ = 503.02, [37M+H]^+^ = 504.79, chloride pattern, Purity = 98.49%. Chiral HPLC 98.80% ee

# **Extended methods for Folch-butanol extraction of lysolipid species from cell media**

Folch extraction: To 1000 µL cell media, add 2000 µL chloroform and 1000 µL methanol to achieve a ratio of 2:1:1 chloroform:methanol:water. Add 10 µL internal standard (LPA 17:0, LPC 19:0, 0.1ng/µL). Vortex (min 15 s) and centrifuge (5 mins, 4200 rpm). Collect the organic phase (lower layer) and dry in a vacuum concentrator (no heat, pressure 2.00 torr). Once dry, store at -20^o^C until completion of second extraction step.

Butanol extraction: To the remaining aqueous phase add 500 μL water and 1000 μL of butanol. Vortex (min 15 s) and centrifuge (5 mins, 4200 rpm). Collect the butanol upper phase and re-extract the lower phase with 500 μL of butanol. Collect the new butanol upper phase and combine with the previous butanol phase. To the combined butanol layers, add 1000 μL of butanol-saturated water (lower layer of 1:1 butanol:water), vortex (min 15 s) and centrifuge (5 mins, 4200 rpm). Collect the upper phase and add 500 μL of water-saturated butanol (upper layer 1:1 butanol:water) to the remaining lower phase. Vortex (min 15 s) and centrifuge (5 mins, 4200 rpm). Collect the upper phase and combine with the previous butanol phase. Dry the combined butanol layers in a vacuum concentrator (no heat, pressure 2.00 torr). After drying, resuspend the butanol extract with the extract from the Folch extraction, in 40 μL of acidified methanol (500 μL of 1M ammonium formate, 580 μL formic acid added to 49.5 mL methanol) and transfer to an LC-MS vial.

A maximum number of 8 samples were prepared at a time and run on the mass spectrometer to avoid issues with sample stability. Repeats were run in a different sample order to the previous runs.

5 µl of sample mix was injected onto a Kinetex EVO C18, 2.6 µm, 100 Å, 100 x 2.1 mm column.

**HPLC solvents**:

Solvent A: 5mM ammonium formate in water + 0.5% formic acid

Solvent B: 5mM ammonium formate in 5% water + 95% acetonitrile + 0.5% formic acid

**HPLC gradient:**

| Time /min | Flow rate ml/min | % solvent B |
| --- | --- | --- |
| 0 | 0.2 | 10 |
| 8 | 0.2 | 10 |
| 13 | 0.2 | 50 |
| 25 | 0.2 | 95 |
| 37 | 0.2 | 95 |
| 37.1 | 0.2 | 10 |
| 40 | 0.2 | 10 |

**Mass spectrometer: Sciex QTRAP 6500 in low mass mode.**

Polarity: Negative.

Mass spectrometer method Parameter Table

CUR: 20.00

TEM: 400.00

GS1: 40.00

GS2: 30.00

CAD: -2.00

IS: -4500.00

DP -200.00

EP -10.00

CE -30.00

CXP -13.00

| Q1 Mass (Da) | Q3 Mass (Da) | Dwell(ms) | ID |
| --- | --- | --- | --- |
| 409.236 | 153 | 30 | LPA 16:0 |
| 407.22 | 153 | 30 | LPA 16:1 |
| 423.252 | 153 | 30 | IS LPA 17:0 |
| 437.267 | 153 | 30 | LPA 18:0 |
| 435.252 | 153 | 30 | LPA 18:1 |
| 433.236 | 153 | 30 | LPA 18:2 |
| 459.252 | 153 | 30 | LPA 20:3 |
| 457.236 | 153 | 30 | LPA 20:4 |
| 455.22 | 153 | 30 | LPA 20:5 |
| 485.267 | 153 | 30 | LPA 22:4 |
| 483.252 | 153 | 30 | LPA 22:5 |
| 481.2 | 153 | 30 | LPA 22:6 |
| 540.331 | 480.31 | 30 | LPC 16:0 |
| 538.315 | 478.294 | 30 | LPC 16:1 |
| 568.362 | 508.34 | 30 | LPC 18:0 |
| 566.346 | 506.33 | 30 | LPC 18:1 |
| 564.331 | 504.31 | 30 | LPC 18:2 |
| 582.378 | 522.36 | 30 | IS LPC 19:0 |
| 590.346 | 530.325 | 30 | LPC 20:3 |
| 588.331 | 528.31 | 30 | LPC 20:4 |
| 586.315 | 526.294 | 30 | LPC 20:5 |
| 616.362 | 556.341 | 30 | LPC 22:4 |
| 614.346 | 554.325 | 30 | LPC 22:5 |
| 612.331 | 552.31 | 30 | LPC 22:6 |

PC is measured here as the formate adduct

- **Fibroblast cell maintenance**

BJ-5ta cells (ATCC Cat#CRL-4001, RRID:CVCL_6573) were cultured a 4:1 mixture of Dulbecco’s medium and Medium 199: 4 parts of DMEM containing 4 mM L-glutamine, 4.5 g/L glucose and 1.5 g/L sodium bicarbonate and 1 part of Medium 199, supplemented with 0.01 mg/mL Hygromycin B and 10% FBS

- **Immunofluorescence**

1x10^4^ cells/well were seeded in 96-well clear bottom, black walled plates. The next day, complete media was replaced by 0.4% FBS media for 6 hours. To verify collagen deposition, we compared 0082T cells to MIA PaCa-2 cancer cells using the previously described Scar-in-a-Jar assay (3,4) which uses Ficoll, a highly branched polysaccharide, to promote ECM deposition. For Scar-in-a-Jar assays, medium was replaced with 0.4% FBS medium containing 35.5 mg/mL Ficoll 70, 25 mg/mL Ficoll 400, and 16.6 µg/mL ascorbic acid. The same conditions without Ficoll were used for the αSMA detection experiment and as negative control for collagen deposition. Where indicated, medium was additionally supplemented with 2 ng/mL TGF-β_1_ (R&D system Cat#240-B). Experiments were performed with 3-4 replicates for each condition. After 3 days, cells were fixed with ice-cold methanol (VWR Chemicals, Cat#20864.320). Immunodetection was performed using antibodies against ɑSMA (Sigma-Aldrich Cat#A5228, RRID:AB_262054) or collagen I (Sigma-Aldrich Cat# C2456, RRID:AB_476836) at 1:500 dilution for 1.5 hours at RT, or overnight at 4^o^C. Cells were then incubated with AlexaFluor488 or AlexaFluor568 (Invitrogen Cat# A11001 and A11004) secondary antibodies at 1:500 and HCS nuclear mask (Invitrogen Cat# H10325) at 1:2000 for 1 hour at room temperature. Images were acquired on the CellInsight CX5 High Content Screening platform (ThermoScientific). At least 16 fields of views were acquired per well at 10X or 20X magnification. aSMA staining was quantified using the ‘Target Activation advanced v4.1’ algorithm of the Cellomics HCS Studio analysis software (version 6.6.1) to determine cell number and fluorescent intensity per well.

- ***Conditioned media generation***

For concentrated condition media, 4x10^5^ 0082T or PANC-1 cells/well were seeded in a 6-well plate in complete media. The next day, the wells were washed twice with SF DMEM. 3 mL/well of SF DMEM + 0.5% fatty acid-free bovine serum albumin (FAF BSA) either with or without 0.1% DMSO or 12 µM of compounds were added to wells. Conditioned media (CM) was collected post 48 or 72 hours and centrifuged at 300xg for 5 minutes to remove cell debris. 3 kDa cut-off ultracentrifuge tubes were used to generate concentrated CM. As established in (5) SF DMEM contained 1 mM glucose, and 0.5 mM glutamax.

- ***Cell growth assays***

MIA PaCa-2 and PANC-1 were seeded at 5x10^3^ cells per well in a 96-well clear bottom black walled microplate. The next day, media was replaced with DMEM supplemented with 0.5% FAF BSA. After 24 hours, PDAC cells were treated as indicated and placed in the Incucyte for live-cell imaging. 0082T CAF cells were seeded at 5x10^3^ cells per well in a 96-well plate. The following day, 0082T cells were treated with treatment conditions as indicated and either incubated for 48 hours at 37°C and 5% CO2 for a readout with CellTiter-Glo (Promega) or live-imaged with the Incucyte. Incucyte Cytotox dye (Sartorius Cat#4633) was used to detect cell death at 1:4000 and Incucyte Nuclight red (Sartorius Cat#4717) was used at 1:500. 18:1 LPA (Merck, Cat#857130P) was resuspended in PBS with 0.1% fatty acid free BSA for a 10 mM stock concentration. The ATX inhibitors, IOA-289 (2), PF-8380 (6) , HA-130 (7) (Sigma-Aldrich Cat# 189511-10MG), GLPG1690 (8) were used at 12 µmol/L. The controls DMSO and Staurosporine or STS (Sigma-Aldrich Cat# S4400) were used at 0.1% and 1 µmol/L respectively.

**References**

1. Schadt O, Dorsch D, Stieber F, Blaukat A. 2-oxo-3-benzylbenzoxazol-2-one derivatives and related compounds as met kinase inhibitors for the treatment of tumours. US2010280030A1, 2010. p. 32.

2. Shah P, Cheasty A, Foxton C, Raynham T, Farooq M, Gutierrez IF, et al. Discovery of potent inhibitors of the lysophospholipase autotaxin. Bioorg Med Chem Lett. 2016 Nov 15;26(22):5403–10.

3. Martufi M, Good RB, Rapiteanu R, Schmidt T, Patili E, Tvermosegaard K, et al. Single-Step, High-Efficiency CRISPR-Cas9 Genome Editing in Primary Human Disease-Derived Fibroblasts. CRISPR J. 2019 Feb;2(1):31–40.

4. Good RB, Eley JD, Gower E, Butt G, Blanchard AD, Fisher AJ, et al. A high content, phenotypic ‘scar-in-a-jar’ assay for rapid quantification of collagen fibrillogenesis using disease-derived pulmonary fibroblasts. BMC Biomed Eng. 2019 Dec;1(1):14.

5. Auciello FR, Bulusu V, Oon C, Tait-Mulder J, Berry M, Bhattacharyya S, et al. A Stromal Lysolipid-Autotaxin Signaling Axis Promotes Pancreatic Tumor Progression. Cancer Discov. 2019 May;9(5):617–27.

6. Gierse J, Thorarensen A, Beltey K, Bradshaw-Pierce E, Cortes-Burgos L, Hall T, et al. A novel autotaxin inhibitor reduces lysophosphatidic acid levels in plasma and the site of inflammation. J Pharmacol Exp Ther. 2010 Jul;334(1):310–7.

7. Albers HMHG, Dong A, Van Meeteren LA, Egan DA, Sunkara M, Van Tilburg EW, et al. Boronic acid-based inhibitor of autotaxin reveals rapid turnover of LPA in the circulation. Proc Natl Acad Sci. 2010 Apr 20;107(16):7257–62.

8. Desroy N, Housseman C, Bock X, Joncour A, Bienvenu N, Cherel L, et al. Discovery of 2-[[2-Ethyl-6-[4-[2-(3-hydroxyazetidin-1-yl)-2-oxoethyl]piperazin-1-yl]-8-methylimidazo[1,2-a]pyridin-3-yl]methylamino]-4-(4-fluorophenyl)thiazole-5-carbonitrile (GLPG1690), a First-in-Class Autotaxin Inhibitor Undergoing Clinical Evaluation for the Treatment of Idiopathic Pulmonary Fibrosis. J Med Chem. 2017 May 11;60(9):3580–90.
